# Supplementary material for: Identifying individuals at high risk for dementia in primary care: Development and validation of the DemRisk risk prediction model using routinely collected patient data
Source: PLoS One. 2024 Oct 4;19(10):e0310712. doi: 10.1371/journal.pone.0310712 (PMC11452046; doi:10.1371/journal.pone.0310712)
Supplement: S1 File — (DOC) [file pone.0310712.s001.DOC]

**S1 File. Additional information**

**Figure A1 Distributions of patient ages for the 60 to 79 cohort based on year-band 1 and on randomly selected year-bands**


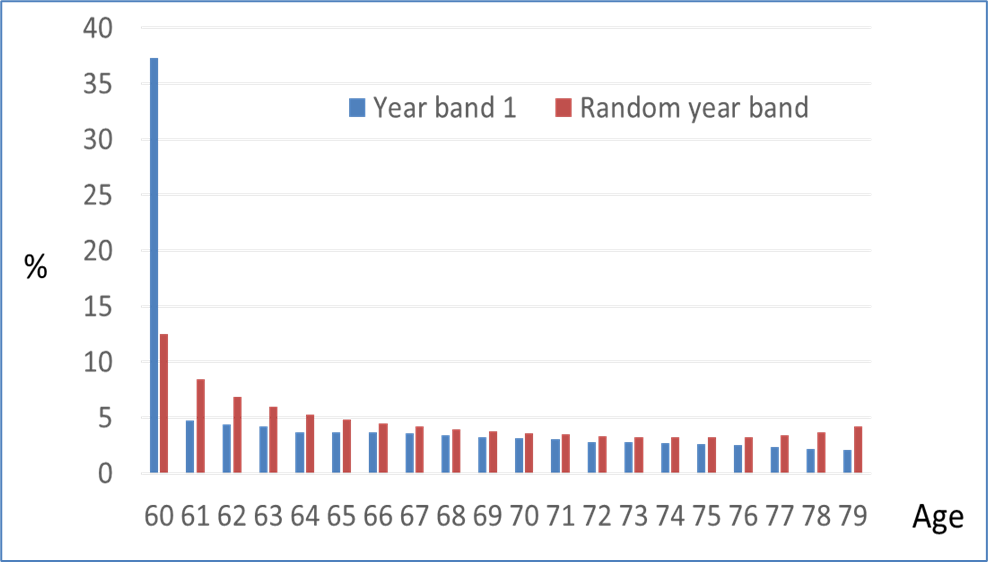


**Figure A2 Calibration plots**


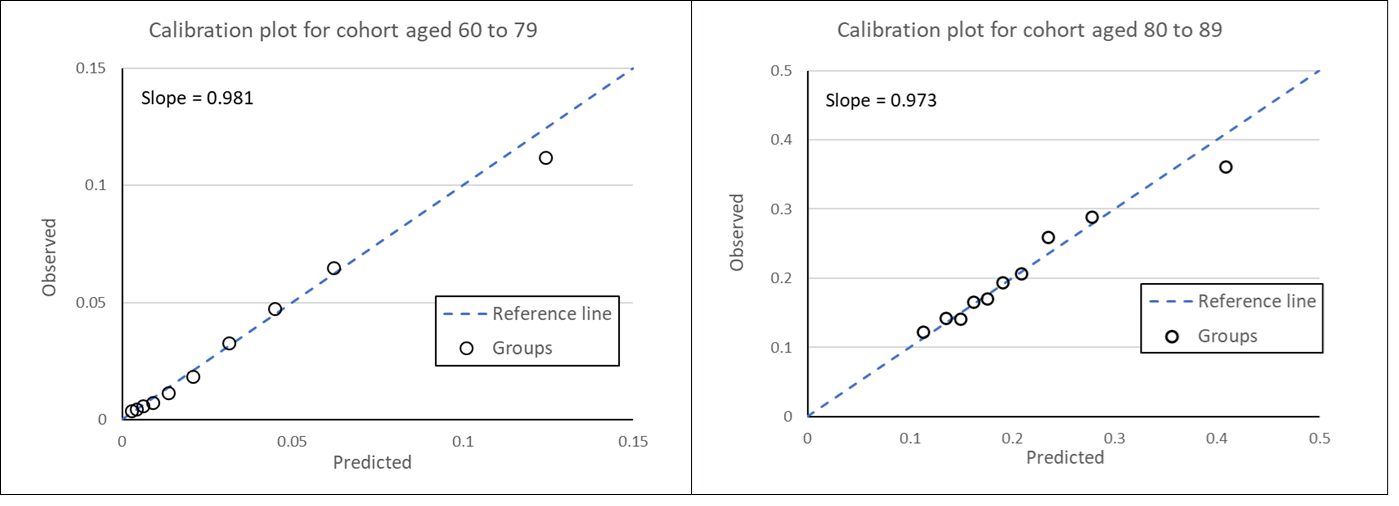


**Table A1. List of candidate predictive factors^a^ identified from the literature along with source papers**

| **Characteristics** | **Tang 2015(1)** | **Ford 2018(2)** | **Ford 2019(3)** | **Other source^b^** |
| --- | --- | --- | --- | --- |
| **Demographics and life-style factors** | | | | |
| Sex | Y |  |  |  |
| Age | y |  |  |  |
| Social deprivation |  |  |  | Walters 2016(4) |
| Smoking | y |  |  |  |
| Alcoholism/alcohol use | y | y | y |  |
| **Medical conditions/symptoms** | | | | |
| Anxiety |  | y | y |  |
| Depression/low mood | y | y | y |  |
| Epilepsy or seizures |  |  | y |  |
| Angina | y |  | y |  |
| Atrial fibrillation | y |  |  |  |
| Coronary bypass surgery | y |  |  |  |
| Cardiomyopathy |  |  |  | Lee 2022(5) |
| Coronary heart disease | y |  |  |  |
| Heart Failure | y |  |  |  |
| Myocardial infarction | y |  |  |  |
| Stroke/TIA | y | y | y |  |
| Cancer |  |  | y |  |
| Diabetes | y | y |  |  |
| Inflammatory conditions, including bowel |  | Y |  |  |
| Chronic renal failure/kidney disease |  | Y |  |  |
| Fracture | y |  | y |  |
| Falls / gait disturbance |  | y | y |  |
| Head Trauma | y |  |  |  |
| Deafness/ear trouble | y |  |  |  |
| Eye Trouble | y |  |  |  |
| Sleep disturbance | y |  |  |  |
| Hyperlipidaemia | y |  |  |  |
| Hypertension | y |  | y |  |
| Hypotension |  | y |  |  |
| **Medications** | | | | |
| Antidepressants |  | y | y |  |
| Antipsychotics |  | y | y |  |
| Benzodiazapines |  |  | y |  |
| Anticonvulsants |  | y |  |  |
| Hypnotics |  | y |  |  |
| Lithium |  | y | y |  |
| Z-drugs |  |  | y |  |
| Aspirin |  | y |  |  |
| Acid Suppressants |  |  |  | Wang 2022(6), Chen 2020(7) |
| NSAIDS |  | y |  |  |
| Opioids |  |  |  | Dublin 2015(8) |
| Statins and lipid lowering drugs |  |  |  | Zhang 2018(9), Chu 2018(10) |
| Antihypertensives | y |  |  |  |
| Anticholinergic drugs |  |  |  | Richardson 2018(11), Coupland 2019(12) |
| Count of medications |  | y |  |  |
| **Service interactions** | | | | |
| Social services referral |  |  | y |  |
| A&E attendance |  |  | y |  |
| 3rd party consultations |  |  | y |  |
| GP home visits |  |  | y |  |
| Missed GP appointments |  |  | y |  |
| **Biometric measurements** | | | | |
| Obesity/BMI | y | y |  |  |
| Total/HDL Cholesterol | y |  |  |  |
| Blood pressure | y |  | y |  |
| ^a^Factors reported to be statistically significantly related to incident dementia, or appears as a factor in a predictive model  ^b^These sources generally relate to factors first identified since the reviews by Tang and Ford were published | | | | |

**Table A2. Estimated yearly True Positive and False Negative rates for patients aged 60-79 years, modelled to account for censoring and assuming annual risk assessments**

| **Threshold for high risk** | **True Positive %** | **False Negative %** |
| --- | --- | --- |
| 3% | 6.3 | 1.2 |
| 4% | 7.4 | 1.5 |
| 5% | 8.7 | 1.8 |
| 10% | 16.3 | 2.5 |
| 15% | 23.5 | 2.8 |
| 20% | 30.4 | 2.9 |
| 25% | 36.8 | 2.9 |

**Table A3. Age 60-79 validation cohort, fit indices (and 95% confidence intervals) for the DemRisk, DRS and age-only models**

| **Model** | **Harrell’s C** | **Royston D** | **Top 1% precision^a^** | **Top 5% precision^a^** |
| --- | --- | --- | --- | --- |
| DemRisk reduced model | 0.781  (0.776 to 0.786) | 1.74  (1.70 to 1.78) | 36.7% | 22.8% |
| Dementia Risk Score (DRS) | 0.773  (0.768 to 0.779) | 1.63  (1.59 to 1.67) | 26.4% | 19.3% |
| Age-only model | 0.735  (0.729 to 0.741) | 1.28  (1.24 to 1.32) | 16.8% | 11.6% |

^a^Proportion of patients in the top 1%/5% of risk scores with incident dementia within 5 years

**Table A4. Characteristics of development and validation samples of patients aged 80-89 at the index year**

|  | **Development cohort** | | **Validation cohort** | |
| --- | --- | --- | --- | --- |
| Number of GP practices | 235 | | 158 | |
| Total number of patients | 175,131 | | 118,717 | |
| Median length of FU | 1.81 | | 1.85 | |
| Interquartile range of FU | (0.7, 3.83) | | (0.68, 3.83) | |
| Number of incident dementia diagnoses (within 5 years) | 15,994 | | 11,078 | |
| Total person-years at risk | 397,710 | | 270,085 | |
|  | **N (%)** | **Mean (SD)** | **N (%)** | **Mean (SD)** |
| **Demographic and lifestyle factors** | | | | |
| Sex | - |  | - |  |
| Male | 70,977 (40.5) | - | 47,815 (40.3) | - |
| Female | 104,154 (59.5) | - | 70,902 (59.7) | - |
| Age | - | 83.2 (2.92) | - | 83.2 (2.92) |
| Practice IMD (quintiles) |  |  |  |  |
| 1 (lowest deprivation) | 28,103 (16.1) | - | 21,003 (17.7) | - |
| 2 | 34,220 (19.5) | - | 24,348 (20.5) | - |
| 3 | 36,268 (20.7) | - | 23,589 (19.9) | - |
| 4 | 34,847 (19.9) | - | 28,058 (23.6) | - |
| 5 (highest deprivation) | 41,693 (23.8) | - | 21,719 (18.2) | - |
| Smoking status |  |  |  |  |
| Never smoked | 65,289 (37.3) | - | 44,290 (37.3) | - |
| Ex-smoker | 82,656 (47.2) | - | 56,157 (47.3) | - |
| Current Smoker | 20,664 (11.8) | - | 14,602 (12.3) | - |
| Not recorded | 6,522 (3.7) | - | 3,668 (3.1) | - |
| Heavy drinking/alcohol problem ever | 13,652 (7.8) | - | 9.095 (7.7) | - |
| **Medical conditions (ever recorded)** | | | | |
| Anxiety | 30,576 (17.5) | - | 19,852 (16.7) | - |
| Depression | 29,807 (17.0) | - | 19,0663 (16.1) | - |
| Epilepsy | 2,563 (1.5) | - | 1,629 (1.4) | - |
| Angina | 24,955 (14.3) | - | 16,697 (14.1) | - |
| Atrial fibrillation | 23,578 (13.5) | - | 16,011 (13.5) | - |
| Coronary bypass surgery | 5,644 (3.2) | - | 3,633 (3.1) | - |
| Cardiomyopathy | 677 (0.4) | - | 462 (0.4) | - |
| Coronary Heart Disease | 56,036 (32.0) | - | 38,716 (32.6) | - |
| Heart failure | 15,825 (9.0) | - | 10,859 (9.2) | - |
| Myocardial infarction | 15,862 (9.1) | - | 10,500 (8.8) | - |
| Stroke/TIA | 23,252 (13.3) | - | 15,808 (13.3) | - |
| Cancer (any) | 47,015 (26.9) | - | 31,149 (26.2) | - |
| Diabetes | 26,636 (15.2) | - | 18,055 (15.2) | - |
| Irritable bowel disease | 2,775 (1.6) | - | 1,828 (1.5) | - |
| Renal disease/failure | 40,389 (23.1) | - | 28,053 (23.6) | - |
| Fracture (any) | 45,963 (26.2) | - | 29,943 (25.2) | - |
| Gait disturbance | 7,781 (4.4) | - | 4,920 (4.1) | - |
| Mild traumatic head injury (incl. concussion) | 7,445 (4.3) | - | 4,747 (4.0) | - |
| Major trauma head injury | 8,675 (5.0) | - | 5,546 (4.7) | - |
| Hearing impairment | 49,648 (28.4) | - | 34,238 (28.8) | - |
| Sight impairment | 8,534 (4.9) | - | 5,829 (4.9) | - |
| Sleep disturbance | 31,894 (18.2) | - | 20,537 (17.3) | - |
| Hyperlipidaemia | 37,700 (21.5) | - | 27,685 (23.3) | - |
| Hypertension | 107,486 (61.4) | - | 72,966 (61.5) | - |
| Hypotension | 6,943 (4.0) | - | 4,202 (3.5) | - |
| **Prescribed medications (ever prescribed)** | | | | |
| Anti-depressants |  |  |  |  |
| Selective serotonin reuptake inhibitor | 27,614 (15.8) | - | 17,670 (14.9) | - |
| Tricyclic antidepressant | 44,397 (25.4) | - | 28,198 (23.8) | - |
| Other antidepressant | 7,964 (4.6) | - | 5,001 (4.2) | - |
| Antipsychotics | 43,556 (24.9) | - | 27,879 (23.5) | - |
| Benzodiazepines | 42,755 (24.4) | - | 27,990 (23.6) | - |
| Hypnotics | 51,468 (29.4) | - | 33,666 (28.4) | - |
| Mood stabilisers | 6,771 (3.9) | - | 4,618 (3.9) | - |
| Z-drugs | 18,217 (10.4) | - | 11,657 (9.8) | - |
| Aspirin | 87,260 (49.8) | - | 58,584 (49.4) | - |
| H2 receptor antagonists | 38,697 (22.1) | - | 24,908 (21.0) | - |
| Proton pump primers | 83,821 (47.9) | - | 56,479 (47.6) | - |
| NSAIDs | 120,144 (68.6) | - | 79,574 (67.0) | - |
| Opioids | 34,982 (20.0) | - | 23,328 (19.7) | - |
| Statins | 80,165 (45.8) | - | 54,650 (46.0) | - |
| Lipid lowering medication (including statins) | 81,102 (46.3) | - | 55,296 (46.6) | - |
| Anti-hypertensives | 137,907 (78.8) | - | 92,772 (78.2) | - |
| Any anticholinergic | 116,100 (66.3) | - | 76,716 (64.6) | - |
| Anticholinergic burden score over last year |  | 0.84 (1.61) |  | 0.81 (1.57) |
| Polypharmacy count over last year |  | 5.58 (4.85) |  | 5.49 (4.80) |
| **Service interactions** | | | | |
| Ever received social care | 1,531 (0.9) | - | 909 (0.8) | - |
| N of visits to A&E in last year | - | 0.19 (0.56) | - | 0.19 (0.57) |
| N of GP visits with a third party in last year | - | 0.08 (0.52) | - | 0.08 (0.50) |
| N of GP home visits in last year | - | 0.53 (2.24) | - | 0.56 (2.60) |
| N of DNAs in last year | - | 0.11 (0.43) | - | 0.12 (0.44) |
| **Biometric measures** | | | | |
| BMI ever recorded | 154,045 (88.0) | - | 105,964 (89.3) |  |
| Most recently recorded BMI | - | 26.2 (4.7) | - | 26.1 (4.7) |
| Total serum cholesterol ever recorded | 137,603 (78.6) | - | 93,892 (79.1) |  |
| Most recent total serum cholesterol (mmol/L)^a^ | - | 4.9 (1.2) | - | 4.9 (1.2) |
| Blood pressure ever recorded | 170,854 (97.6) | - | 115,074 (976.9) | - |
| Most recent systolic BP (mmHg)^a^ | - | 140.6 (16.2) | - | 140.7 (16.2) |
| Most recent diastolic BP (mmHg)^a^ | - | 76.0 (8.9) | - | 76.0 (8.8) |
| Most recent pulse pressure(mmHg)^a^ | - | 64.6 (14.0) | - | 64.7 (14.0) |
| DNA, Did Not Attend scheduled primary care appointment  ^a^Mean within the most recent year-band with recorded values | | | | |

**Table A5. Summary of univariable analyses of predictive factors, development cohort age 80-89**

|  | **Hazard ratio** | **95% CI** |  |
| --- | --- | --- | --- |
| **Demographic and lifestyle factors** | | | |
| Sex (Female) | 1.220^a^ | 1.180 to 1.262 |  |
| Age - 60 | 1.061 | 1.041 to 1.081 |  |
| (Age - 60)^2 | 0.996 | 0.994 to 0.998 |  |
| All subsequent factors are controlled for age and sex |  |  |  |
| Calendar year (-2005) | 0.974 | 0.967 to 0.981 |  |
| Practice IMD quintile |  |  |  |
| 1 Lowest deprivation | Ref |  |  |
| 2 | 0.982 | 0.895 to 1.078 |  |
| 3 | 1.028 | 0.936 to 1.130 |  |
| 4 | 1.051 | 0.960 to 1.152 |  |
| 5 highest deprivation | 1.172 | 1.066 to 1.289 |  |
| Smoking status, most recent^a^ |  |  |  |
| Non-smoker | Ref |  |  |
| Ex-smoker | 1.009 | 0.971 to 1.046 |  |
| Current smoker | 1.095 | 1.036 to 1.153 |  |
| Heavy drinking/alcohol problem ever | 0.975 | 0.912 to 1.042 |  |
| **Medical conditions (ever recorded)** | | | |
| Anxiety | 1.202 | 1.150 to 1.257 |  |
| Depression | 1.338 | 1.285 to 1.393 |  |
| Epilepsy | 1.337 | 1.184 to 1.511 |  |
| Angina | 1.041 | 0.990 to 1.094 |  |
| Atrial fibrillation | 1.195 | 1.141 to 1.252 |  |
| Coronary bypass surgery | 1.058 | 0.956 to 1.171 |  |
| Cardiomyopathy | 0.913 | 0.667 to 1.249 |  |
| Coronary Heart Disease | 1.037 | 0.997 to 1.078 |  |
| Heart failure | 1.128 | 1.063 to 1.197 |  |
| Myocardial infarction | 1.140 | 1.071 to 1.213 |  |
| Stroke/TIA | 1.441 | 1.381 to 1.503 |  |
| Cancer (any) | 0.926 | 0.890 to 0.964 |  |
| Diabetes | 1.179 | 1.126 to 1.234 |  |
| Irritable bowel disease | 1.024 | 0.906 to 1.157 |  |
| Renal disease/failure | 1.002 | 0.961 to 1.045 |  |
| Fracture (any) | 1.203 | 1.151 to 1.257 |  |
| Gait problems | 1.301 | 1.220 to 1.388 |  |
| Mild traumatic head injury (incl. concussion) | 1.396 | 1.311 to 1.487 |  |
| Major traumatic head injury | 1.386 | 1.306 to 1.472 |  |
| Hearing impairment | 0.995 | 0.962 to 1.029 |  |
| Sight impairment | 1.187 | 1.109 to 1.269 |  |
| Sleep disturbance | 1.046 | 0.988 to 1.107 |  |
| Hyperlipidaemia | 0.965 | 0.924 to 1.008 |  |
| Hypertension | 0.901 | 0.867 to 0.937 |  |
| Hypotension | 1.292 | 1.195 to 1.397 |  |
| **Prescribed medications (ever prescribed)** | | | |
| Anti-depressants |  |  |  |
| Selective serotonin reuptake inhibitor | 1.593 | 1.528 to 1.661 |  |
| Tricyclic antidepressant | 1.116 | 1.073 to 1.160 |  |
| Other antidepressant | 1.660 | 1.550 to 1.779 |  |
| Antipsychotic | 1.163 | 1.118 to 1.210 |  |
| Benzodiazepines | 1.047 | 1.006 to 1.089 |  |
| Hypnotics | 1.080 | 1.040 to 1.121 |  |
| Mood stabiliser | 1.299 | 1.217 to 1.386 |  |
| Z-drugs | 1.166 | 1.096 to 1.240 |  |
| Aspirin | 1.179 | 1.138 to 1.222 |  |
| H2 receptor antagonists | 0.954 | 0.914 to 0.996 |  |
| Proton pump primers | 0.969 | 0.938 to 1.002 |  |
| NSAIDs (excluding aspirin) | 0.901 | 0.864 to 0.939 |  |
| Opioids | 1.038 | 0.994 to 1.083 |  |
| Statins | 1.029 | 0.991 to 1.068 |  |
| Lipid lowering medication (including statins) | 1.032 | 0.995 to 1.071 |  |
| Antihypertensive | 1.028 | 0.980 to 1.078 |  |
| Any anticholinergic | 1.121 | 1.074 to 1.171 |  |
| Anticholinergics burden over last year (square root) | 1.144 | 1.118 to 1.171 |  |
| Polypharmacy count over last year | 1.018 | 1.014 to 1.021 |  |
| **Service interactions** | | | |
| Ever received social care | 1.796 | 1.517 to 2.126 |  |
| Number of A&E visits in last year (square root) | 1.453 | 1.398 to 1.510 |  |
| N of consultations including a third party in last year (square root) | 1.581 | 1.470 to 1.700 |  |
| N of home visits in last year (square root) | 1.354 | 1.308 to 1.401 |  |
| N of DNAs in last year (square root) | 1.560 | 1.492 to 1.631 |  |
| **Biometric measures** | | | |
| Most recent BMI (square root)^a^ | 0.667 | 0.640 to 0.694 |  |
| Most recent mean Serum Cholesterol^ab^ | 0.984 | 0.969 to 0.999 |  |
| Most recent mean Systolic BP/20^abc^ | 0.869 | 0.850 to 0.887 |  |
| Most recent mean Diastolic BP/20^abc^ | 0.948 | 0.917 to 0.980 |  |
| Most recent pulse pressure(mmHg)^abc^ | 0.848 | 0.827 to 0.869 |  |
| ^a^Pooled across 10 datasets with missing data values imputed  ^b^Mean within the most recent year-band with recorded values  ^c^Rescaled by dividing by 20 | | |  |

**Table A6. Age 80-89 cohort, fit statistics for the full and reduced models based on the development and validation cohorts**

| **Model** | **Dataset** | **Harrell’s C** | **Royston D** | **Top 1% precision^a^** | **Top 5% precision^a^** | **Calibration slope** |  |
| --- | --- | --- | --- | --- | --- | --- | --- |
| Full model | Development cohort | 0.646  (0.640 - 0.651) | 0.822  (0.788 - 0.856) | 81.0% | 69.4% | NA |  |
| Reduced model | Development cohort | 0.635  (0.629- 0.640) | 0.734  (0.701 - 0.766) | 82.0% | 71.9% | NA |  |
| Reduced model | Validation cohort | 0.637  (0.630 - 0.643) | 0.737  (0.700 - 0.774) | 78.6% | 71.0% | 0.973  (0.919 – 1.027) |  |
| ^a^Proportion of patients in the top 1%/5% of risk scores receiving a diagnosis of dementia within 5 years | | | | | | | |

**Table A7. Estimated yearly True Positive and False Negative rates for patients aged 80-89 years, modelled to account for censoring and assuming annual risk assessments**

| **Threshold for high risk** | **True Positive %** | **False Negative %** |
| --- | --- | --- |
| 10% | 20.7 | 9.2 |
| 15% | 23.1 | 13.0 |
| 20% | 28.3 | 15.6 |
| 25% | 34.1 | 17.2 |
| 30% | 39.8 | 18.3 |
| 40% | 50.7 | 19.5 |
| 50% | 60.9 | 20.1 |

**Table A8. Age 80-89 validation cohort, fit indices (and 95% confidence intervals) for the DemRisk, DRS and age-only models**

| **Model** | **Harrell’s C** | **Royston D** | **Top 1% precision^a^** | **Top 5% precision^a^** |
| --- | --- | --- | --- | --- |
| DemRisk reduced model | 0.637  (0.630 to 0.643) | 0.737  (0.700 to 0.774) | 78.6% | 71.0% |
| Dementia Risk Score (DRS) | 0.608  (0.602 to 0.614) | 0.594  (0.558 to 0.629) | 67.0% | 58.2% |
| Age-only model | 0.533  (0.527 to 0.539) | 0.217  (0.185 to 0.249) | 40.1% | 36.7% |

^a^Proportion of patients in the top 1%/5% of risk scores with incident dementia within 5 years

**Computation of risk scores for patients with missing data values**

Data was missing for some patients on two of the risk factors in our final models, BMI and systolic blood pressure. Numbers of missing values were fairly low: 10% of patients in the 60-79 cohort had data missing on one or both of these factors; for the 80-89 cohort it was 12%.

Our models are designed to be automated within the record systems of primary care practices, to produce risk scores for individual patients “on the fly” as and when needed. For cases with missing risk factor data however, that is not possible until the missing information has been obtained and entered into the EHR. This can be time-consuming, delay the estimation of risk scores, and result in patients being excluded from routine screening. An alternative is to impute the missing values and derive a risk score by substituting the imputed values into the risk model. Ideally, the missing values would be imputed using the same fully conditional specification (FCS) procedure that was used to address missingness during the model development and validation stages. However, when deployed in daily practice it is would not be practical (nor perhaps even possible) to apply FCS to new patients with missing measures (i.e. patients not part of the development and validation samples) without a great deal of associated programming.(13) In lieu of that, we investigated whether a simple imputation method could be used to generate a reasonably accurate risk score, perhaps as a stop-gap until the missing data could be obtained.

We explored a variation on what is commonly termed “mean imputation”, whereby missing values are replaced by the mean value across the rest of the sample (i.e. the non-missing cases). However, we instead utilised the mean of the values imputed using FCS, based on the subset of patients in the development sample with a missing measure. We first averaged the FCS imputations across the 10 imputation datasets and then across the patients, to produce a single imputation value that can substitute for a missing data value in new patients. We did this separately for BMI and for BP, within the younger and older age cohorts . We based the mean on the FCS-generated imputations, rather than on the observed values for non-missing cases, because the FCS imputations take into account any systematic differences that might exist between patients with and without missing values. To assess how this approach might perform in practice, we used our “mean-FCS” imputation method to compute risk scores (in the form of probabilities) for all cases in the validation sample with missing data. We then compared these scores to those previously obtained for the same patients using the patient-specific FCS imputations, where the latter represents the preferred – even if unobtainable – imputations for each individual patient.

The age 60-79 validation cohort included 45,575 patients with missing BMI and/or BP measures. The mean-FCS imputed value was 26.0 for BMI and 136 for systolic BP. Mean risk scores and ranges were very similar under both imputation methods (Table A9). Differences between the methods in estimated risk scores for individual patients were all <9% and exceeded 5% for only 33 (0.07%) of patients. The two methods also produced very similar classifications of the patients into high- versus low-risk groups, with the percentage of disagreements being less than 1% at threshold values of 10% or higher (Table A10)

**Table A9. Age 60-79 validation cohort. Comparison of risk scores using two imputation methods**

| **Form of imputation** | **Number of patients with missing BMI or BP** | **Mean risk score** | **Range** | **% of differences>5%** |
| --- | --- | --- | --- | --- |
| Patient-specific FCS imputations | 45,575 | 2.75% | 0.1% to 99.0% | NA |
| Mean-FCS imputations | 45,575 | 2.71% | 0.1% to 97.8% | NA |
| Difference | 45,575 | -0.04% | -7.8% to 8.7% | 0.07% (n=33) |

**Table A10. Age 60-79 validation cohort. Comparison of risk group classification of patients with missing BMI and/or BP measures under patient-specific FCS and mean-FCS imputation**

| **Threshold for high risk** | **Patients classified as low risk under both imputation methods** | **Patients classified as high risk under both imputation methods** | **Patients classified as low risk under patient-specific FCS and high risk under mean-FCS imputation** | **Patients classified as high risk under patient-specific FCS and low risk under mean-FCS imputation** |
| --- | --- | --- | --- | --- |
| 5% | 37,060 (81.3%) | 7,067 (15.5%) | 387 (0.9%) | 1,061 (2.3%) |
| 10% | 43,988 (96.5%) | 1,311 (2.9%) | 108 (0.2%) | 168 (0.4%) |
| 15% | 44,919 (98.6%) | 526 (1.2%) | 63 (0.1%) | 67 (0.2%) |
| 20% | 45,235 (99.3%) | 266 (0.6%) | 31 (0.07%) | 43 (0.09%) |
| 25% | 45,396 (99.6%) | 150 (0.3%) | 15 (0.03%) | 14 (0.03%) |

The age 80-89 validation cohort included 14,183 patients with missing BMI and/or BP measures. The overall mean-FCS imputed values (averaged across all patients in the development sample) were 24.8 for BMI and 143 for systolic BP. Mean risk scores and ranges were very similar under both imputation methods (Table A11). Differences between the methods in estimated risk scores for individual patients were all <9% and exceeded 5% for only 96 (0.7%) of patients. Agreement between the two methods on classification of patients into high- versus low-risk groups was somewhat lower for this older age-group, but at threshold scores of 25% or more the methods disagreed on less than 3% of cases. (Table A12)

**Table A11. Age 80-89 validation cohort. Comparison of risk scores using two imputation methods**

| **Form of imputation** | **Number of patients with missing BMI or BP** | **Mean risk score** | **Range** | **% of differences>5%** |
| --- | --- | --- | --- | --- |
| Patient-specific FCS imputations | 14,183 | 21.1% | 7.0% to 96.9% | NA |
| Mean-FCS imputations | 14,183 | 20.8% | 7.2% to 97.4% | NA |
| Difference | 14,183 | -0.2% | -8.8% to 7.9% | 0.7% (n=96) |

**Table A12. Age 80-89 validation cohort. Comparison of risk group classification of patients with missing BMI and/or BP measures under patient-specific FCS and mean-FCS imputation**

| **Threshold for high risk** | **Patients classified as low risk under both imputation methods** | **Patients classified as high risk under both imputation methods** | **Patients classified as low risk under patient-specific FCS and high risk under mean-FCS imputation** | **Patients classified as high risk under patient-specific FCS and low risk under mean-FCS imputation** |
| --- | --- | --- | --- | --- |
| 20% | 8,351 (58.9%) | 4,958 (35.0) | 271 (1.9%) | 603 (4.3%) |
| 25% | 11,097 (78.2%) | 2,666 (18.8%) | 148 (1.0%) | 272 (1.9%) |
| 30% | 12,383 (87.3%) | 1,520 (10.7%) | 89 (0.6%) | 191 (1.4%) |
| 40% | 13,527 (95.4%) | 529 (3.7%) | 38 (0.3%) | 89 (0.6%) |

In summary, the use of mean-FCS imputation results in risk scores changing by 5% or more for just 0.07% of patients aged 60 to 79 and 0.7% of those aged 80 to 89, compared to use of the ideal patient-specific FCS method. The impact of this on the classification of patients into high- and low-risk groups is more pronounced and depends upon the threshold score used to define high risk, though in most scenarios less than 2% of patients change from low- to high-risk (or vice-versa) as a result. One reason why risk scores and classifications differ only a little between methods, is because most of the risk score is contributed by other risk factors in the model that are not subject to missing values. To apply the method in practice simply requires patients aged 60 to79 with a missing BMI measure to have this assigned as 26.0, and a missing systolic BP assigned as 136, when computing the risk score. For patients aged 80 to 89 the corresponding values are 24.8 and 143.

**Calculation of a 5-year DemRisk risk score for patients aged 60 to 79 years**

Example for an individual patient, age 67

Baseline survival prob at 5 years S = 0.9525

| Predictive factor | Patient data^a^ | Transformation where required | Transformed value (T)^b^ | Weight (W)^c^ | Product (T x W) | |  |  |
| --- | --- | --- | --- | --- | --- | --- | --- | --- |
| **Demographic and lifestyle factors** | | | | | | | | |
| Age in years | 67 | age in years - 60 | 7.00 | 0.268 | 1.876 | |  |  |
|  |  | (age in years - 60)^2 | 49.00 | -0.006 | -0.294 | |  |  |
| **Practice area deprivation quintile** | | | | | | | | |
| Quintile 1 (least deprived) | - |  | 0.00 | 0 | 0 | |  |  |
| Quintile 2 | - |  | 0.00 | 0.001 | 0 | |  |  |
| Quintile 3 | - |  | 0.00 | 0.012 | 0 | |  |  |
| Quintile 4 | - |  | 0.00 | 0.071 | 0 | |  |  |
| Quintile 5 | Yes |  | 1.00 | 0.221 | 0.221 | |  |  |
| **Medical conditions** | | | | | | | | |
| History of depression | Yes |  | 1.00 | 0.121 | 0.121 | |  |  |
| History of stroke | Yes |  | 1.00 | 0.486 | 0.486 | |  |  |
| History of diabetes |  |  | 0.00 | 0.304 | 0 | |  |  |
| History of epilepsy |  |  | 0.00 | 0.435 | 0 | |  |  |
| History of gait problems | Yes |  | 1.00 | 0.414 | 0.414 | |  |  |
| History of major head injury | - |  | 0.00 | 0.307 | 0 | |  |  |
| **Treatments** | | | | | | | | |
| Use of SSRIs | Yes |  | 1.00 | 0.381 | 0.381 | |  |  |
| Use of TCAs |  |  | 0.00 | -0.095 | 0 | |  |  |
| Use of other antidepressants |  |  | 0.00 | 0.190 | 0 | |  |  |
| Use of mood stabilisers |  |  | 0.00 | 0.227 | 0 | |  |  |
| Use of NSAIDs | Yes |  | 1.00 | -0.158 | -0.158 | |  |  |
| Total anticholergenic use | 10 | Square-root | 3.16 | 0.136 | 0.430 | |  |  |
| **Service interactions** | | | | | | | | |
| Has received social services |  |  | 0.00 | 0.782 | 0.000 | |  |  |
| Number of A&E visits in previous 12 months | 1 | Square-root | 1.00 | 0.255 | 0.255 | |  |  |
| Number of GP consultations involving third party in previous 12 months | 3 | Square-root | 1.73 | 0.333 | 0.577 | |  |  |
| Number of GP home visits in previous 12 months | 0 | Square-root | 0.00 | 0.240 | 0.000 | |  |  |
| Number of missed GP appointments in previous 12 months | 2 | Square-root | 1.41 | 0.408 | 0.577 | |  |  |
| **Biometric measures** | | | | | | | | |
| BMI value^d^ | 23 | Square-root | 4.80 | -0.486 | -2.331 | |  |  |
| Systolic blood pressure (mmHg)^d^ | 120 | divide by 20 | 6.00 | -0.050 | -0.300 | |  |  |
| **Total = R =** | | | | | | **2.255** | |  |
| The baseline survival probability for the 60-79 age group is S = 0.9525  The raw score, R, is converted into a percentage risk as: % risk = (1 - S^exp(R)^) x 100% = 37%. Overall, 37% of patients with the above pattern of data are expected to acquire a diagnosis of dementia within 5 years. | | | | | | | |  |
| ^a^A blank in this column implies No  ^b^A Yes in column 2 is coded as 1 here; a No or blank is coded as 0  ^c^ The weight is the raw coefficient from the Cox regression | | | | | | | |  |

**Calculation of a 5-year DemRisk risk score for patients aged 80 to 89 years**

Example for an individual patient, age 84

Baseline survival prob at 5 years S = 0.1207

| Predictive factor | Patient data^a^ | Transformation where required | Transformed value (T)^b^ | Weight (W)^c^ | Product (T x W) | |
| --- | --- | --- | --- | --- | --- | --- |
| **Demographic and lifestyle factors** | | | | | | |
| Female sex | 1 |  | 1 | 0.163 | 0.163 | |
| Age in years | 84 | age in years - 80 | 4.00 | 0.046 | 0.184 | |
|  |  | (age in years - 80)^2 | 16.00 | -0.004 | -0.064 | |
| **Practice area deprivation quintile** | | | | | | |
| Quintile 1 (least deprived) | 1 |  | 1.00 | 0 | 0 | |
| Quintile 2 |  |  | 0.00 | -0.020 | 0 | |
| Quintile 3 |  |  | 0.00 | 0.016 | 0 | |
| Quintile 4 |  |  | 0.00 | 0.039 | 0 | |
| Quintile 5 |  |  | 0.00 | 0.126 | 0 | |
| **Medical conditions** | | | | | | |
| History of stroke |  |  | 0.00 | 0.241 | 0 | |
| History of diabetes | 1 |  | 1.00 | 0.151 | 0.151 | |
| History of gait problems | 1 |  | 1.00 | 0.160 | 0.160 | |
| History of major head injury |  |  | 0.00 | 0.230 | 0 | |
| **Treatments** | | | | | | |
| Use of SSRIs |  |  | 0.00 | 0.320 | 0 | |
| Use of TCAs |  |  | 0.00 | -0.079 | 0 | |
| Use of other antidepressants |  |  | 0.00 | 0.177 | 0 | |
| Use of NSAIDs | 1 |  | 1.00 | -0.145 | -0.145 | |
| Total anticholergenic use | 5 | Square-root | 2.24 | 0.047 | 0.105 | |
| **Service interactions** | | | | | | |
| Has received social services | 1 |  | 1.00 | 0.302 | 0.302 | |
| Number of A&E visits in previous 12 months | 0 | Square-root | 0.00 | 0.179 | 0.000 | |
| Number of GP consultations involving third party in previous 12 months | 3 | Square-root | 1.73 | 0.241 | 0.000 | |
| Number of GP home visits in previous 12 months | 0 | Square-root | 0.00 | 0.211 | 0.000 | |
| Number of missed GP appointments in previous 12 months | 1 | Square-root | 1.00 | 0.321 | 0.321 | |
| **Biometric measures** | | | | | | |
| BMI value^d^ | 35 | Square-root | 5.92 | -0.408 | -2.414 | |
| Systolic blood pressure value^d^ | 140 | divide by 20 | 7.00 | -0.085 | -0.595 | |
| **Total = R =** | | | | | | **-1.995** |
| The baseline survival probability for the 80-89 age group is S = 0.1207  The raw score, R, is converted into a percentage risk as: % risk = (1 - S^exp(R)^) x 100% = 25%. Overall, 25% of patients with the above pattern of data are expected to acquire a diagnosis of dementia within 5 years. | | | | | | |
| ^a^A blank in this column implies No  ^b^A Yes in column 2 is coded as 1 here; a No or blank is coded as 0  ^c^ The weight is the raw coefficient from the Cox regression | | | | | | |

References

1. Tang EY, Harrison SL, Errington L, Gordon MF, Visser PJ, Novak G, et al. Current Developments in Dementia Risk Prediction Modelling: An Updated Systematic Review. PLoS One. 2015;10(9):e0136181.

2. Ford E, Greenslade N, Paudyal P, Bremner S, Smith HE, Banerjee S, et al. Predicting dementia from primary care records: A systematic review and meta-analysis. PLOS ONE. 2018;13(3):e0194735.

3. Ford E, Rooney P, Oliver S, Hoile R, Hurley P, Banerjee S, et al. Identifying undetected dementia in UK primary care patients: a retrospective case-control study comparing machine-learning and standard epidemiological approaches. BMC Medical Informatics and Decision Making. 2019;19(1):248.

4. Walters K, Hardoon S, Petersen I, Iliffe S, Omar RZ, Nazareth I, et al. Predicting dementia risk in primary care: development and validation of the Dementia Risk Score using routinely collected data. BMC Medicine. 2016;14(1):6.

5. Lee H, Kim HK, Kim B, Han K, Park JB, Hwang IC, et al. Augmented risk of dementia in hypertrophic cardiomyopathy: A propensity score matching analysis using the nationwide cohort. PLoS One. 2022;17(6):e0269911.

6. Wang H, Tian L, Yan X. No association between acid suppressant use and risk of dementia: an updated meta-analysis. Eur J Clin Pharmacol. 2022;78(3):375-82.

7. Chen LY, Lin HJ, Wu WT, Chen YC, Chen CL, Kao J, et al. Clinical Use of Acid Suppressants and Risk of Dementia in the Elderly: A Pharmaco-Epidemiological Cohort Study. Int J Environ Res Public Health. 2020;17(21).

8. Dublin S, Walker RL, Gray SL, Hubbard RA, Anderson ML, Yu O, et al. Prescription Opioids and Risk of Dementia or Cognitive Decline: A Prospective Cohort Study. J Am Geriatr Soc. 2015;63(8):1519-26.

9. Zhang X, Wen J, Zhang Z. Statins use and risk of dementia: A dose-response meta analysis. Medicine (Baltimore). 2018;97(30):e11304.

10. Chu CS, Tseng PT, Stubbs B, Chen TY, Tang CH, Li DJ, et al. Use of statins and the risk of dementia and mild cognitive impairment: A systematic review and meta-analysis. Sci Rep. 2018;8(1):5804.

11. Richardson K, Fox C, Maidment I, Steel N, Loke YK, Arthur A, et al. Anticholinergic drugs and risk of dementia: case-control study. BMJ. 2018;361:k1315.

12. Coupland CAC, Hill T, Dening T, Morriss R, Moore M, Hippisley-Cox J. Anticholinergic Drug Exposure and the Risk of Dementia: A Nested Case-Control Study. JAMA Intern Med. 2019;179(8):1084-93.

13. Hoogland J, van Barreveld M, Debray TPA, Reitsma JB, Verstraelen TE, Dijkgraaf MGW, et al. Handling missing predictor values when validating and applying a prediction model to new patients. Statistics in medicine. 2020;39(25):3591-607.
