# Supplementary material for: Identifying individuals at high risk for dementia in primary care: Development and validation of the DemRisk risk prediction model using routinely collected patient data
Source: PLoS One. 2024 Oct 4;19(10):e0310712. doi: 10.1371/journal.pone.0310712 (PMC11452046; doi:10.1371/journal.pone.0310712)
Supplement: S2 File — (DOCX) [file pone.0310712.s002.docx]

**S2 File. Codelists**

| **Read codes used to define new cases of dementia** | |
| --- | --- |
| **Read Code** | **Description** |
| E00..00 | Senile and presenile organic psychotic conditions |
| E00..11 | Senile dementia |
| E00..12 | Senile/presenile dementia |
| E000.00 | Uncomplicated senile dementia |
| E001.00 | Presenile dementia |
| E001000 | Uncomplicated presenile dementia |
| E001100 | Presenile dementia with delirium |
| E001200 | Presenile dementia with paranoia |
| E001300 | Presenile dementia with depression |
| E001z00 | Presenile dementia NOS |
| E002.00 | Senile dementia with depressive or paranoid features |
| E002000 | Senile dementia with paranoia |
| E002100 | Senile dementia with depression |
| E002z00 | Senile dementia with depressive or paranoid features NOS |
| E003.00 | Senile dementia with delirium |
| E004.00 | Arteriosclerotic dementia |
| E004.11 | Multi infarct dementia |
| E004000 | Uncomplicated arteriosclerotic dementia |
| E004100 | Arteriosclerotic dementia with delirium |
| E004200 | Arteriosclerotic dementia with paranoia |
| E004300 | Arteriosclerotic dementia with depression |
| E004z00 | Arteriosclerotic dementia NOS |
| Eu00.00 | [X]Dementia in Alzheimer's disease |
| Eu00000 | [X]Dementia in Alzheimer's disease with early onset |
| Eu00011 | [X]Presenile dementia,Alzheimer's type |
| Eu00012 | [X]Primary degen dementia, Alzheimer's type, presenile onset |
| Eu00013 | [X]Alzheimer's disease type 2 |
| Eu00100 | [X]Dementia in Alzheimer's disease with late onset |
| Eu00111 | [X]Alzheimer's disease type 1 |
| Eu00112 | [X]Senile dementia,Alzheimer's type |
| Eu00113 | [X]Primary degen dementia of Alzheimer's type, senile onset |
| Eu00200 | [X]Dementia in Alzheimer's dis, atypical or mixed type |
| Eu00z00 | [X]Dementia in Alzheimer's disease, unspecified |
| Eu00z11 | [X]Alzheimer's dementia unspec |
| Eu01.00 | [X]Vascular dementia |
| Eu01.11 | [X]Arteriosclerotic dementia |
| Eu01000 | [X]Vascular dementia of acute onset |
| Eu01100 | [X]Multi-infarct dementia |
| Eu01111 | [X]Predominantly cortical dementia |
| Eu01200 | [X]Subcortical vascular dementia |
| Eu01300 | [X]Mixed cortical and subcortical vascular dementia |
| Eu01y00 | [X]Other vascular dementia |
| Eu01z00 | [X]Vascular dementia, unspecified |
| Eu02z00 | [X] Unspecified dementia |
| Eu02z11 | [X] Presenile dementia NOS |
| Eu02z12 | [X] Presenile dementia NOS |
| Eu02z13 | [X] Primary degenerative dementia NOS |
| Eu02z14 | [X] Senile dementia NOS |
| Eu02z16 | [X] Senile dementia, depressed or paranoid type |
| Eu04100 | [X]Delirium superimposed on dementia |
| F110.00 | Alzheimer's disease |
| F110000 | Alzheimer's disease with early onset |
| F110100 | Alzheimer's disease with late onset |
| Fyu3000 | [X]Other Alzheimer's disease |

| **ICD-10 codes used to define dementia in the Hospital Episode Statistics dataset** | |
| --- | --- |
| **Code** | **Description** |
| F00 | Dementia in Alzheimer's disease |
| F00.0 | Dementia in Alzheimer's disease with early onset |
| F00.1 | Dementia in Alzheimer's disease with late onset |
| F00.2 | Dementia in Alzheimer's disease, atypical or mixed type |
| F00.9 | Dementia in Alzheimer's disease, unspecified |
| F01 | Vascular dementia |
| F01.0 | Vascular dementia of acute onset |
| F01.1 | Multi-infarct dementia |
| F01.2 | Subcortical vascular dementia |
| F01.3 | Mixed cortical and subcortical vascular dementia |
| F01.8 | Other vascular dementia |
| F01.9 | Vascular dementia, unspecified |
| F03 | Unspecified dementia |
| G30.0 | Alzheimer's disease with early onset |
| G30.1 | Alzheimer's disease with late onset |
| G30.8 | Other Alzheimer's disease |
| G30.9 | Alzheimer's disease, unspecified |
| G31.0 | Circumscribed brain atrophy |
| G31.1 | Senile degeneration of brain, not elsewhere classified |
| G31.8 | Other specified degenerative diseases of nervous system |
| List taken from NAD 2016: National Audit of Dementia 2016. Guidance for acute hospital sites, appendix B. <https://www.rcpsych.ac.uk/pdf/NAD%20guidance%20(updated)%20v0.1.pdf> | |

| **Read codes potentially indicative of cognitive impairment or memory loss** | |
| --- | --- |
| **Read code** | **Description** |
| 2232 | O/E - mentally confused |
| 2232.11 | O/E - confused |
| 2233 | O/E - delirious |
| 2841 | Confused |
| 2841.11 | Confusion |
| 2842 | Disorientated in time |
| 1B1a.00 | Poor auditory sequential memory |
| 1B1A.12 | Memory loss symptom |
| 1B1A.13 | Memory disturbance |
| 1B1Y.00 | Poor visual sequential memory |
| 1S21.00 | Disturbance of memory for order of events |
| 284..00 | O/E - disorientated |
| 28E..00 | Cognitive decline |
| 28G..00 | Forgetful |
| 28H..00 | Mentally vague |
| 29J4.00 | O/E - sensory inattention |
| 3A10.00 | Memory: own age not known |
| 3A20.00 | Memory: present time not known |
| 3A30.00 | Memory: present place not known |
| 3A40.00 | Memory: present year not known |
| 3A50.00 | Memory: own DOB not known |
| 3A60.00 | Memory: present month not known |
| 3A70.00 | Memory: important event not known |
| 3A80.00 | Memory: import.person not known |
| 3A91.00 | Memory: count down unsuccess. |
| 3AA1.00 | Memory: address recall unsucc. |
| 3AE1.00 | GDS level 2 - very mild cognitive decline |
| 3AE2.00 | GDS level 3 - mild cognitive decline |
| 3AE3.00 | GDS level 4 - moderate cognitive decline |
| 3AE4.00 | GDS level 5 - moderately severe cognitive decline |
| 3AE5.00 | GDS level 6 - severe cognitive decline |
| 3AE6.00 | GDS level 7 - very severe cognitive decline |
| 8BIk.00 | Patient forgets to take medication |
| 8HTY.00 | Referral to memory clinic |
| 9Nk1.00 | Seen in memory clinic |
| E030.00 | Acute confusional state |
| E030.11 | Delirium - acute organic |
| E030.12 | Toxic confusional state |
| E030000 | Acute confusional state, post traumatic |
| E030100 | Acute confusional state, of infective origin |
| E030200 | Subacute confusional state, of endocrine origin |
| E030300 | Acute confusional state, of metabolic origin |
| E030400 | Acute confusional state, of cerebrovascular origin |
| E030z00 | Acute confusional state NOS |
| E031.00 | Subacute confusional state |
| E031.11 | Delirium - subacute organic |
| E031000 | Subacute confusional state, post traumatic |
| E031100 | Subacute confusional state, of infective origin |
| E031300 | Subacute confusional state, of metabolic origin |
| E031400 | Subacute confusional state, of cerebrovascular origin |
| E031z00 | Subacute confusional state NOS |
| E042.00 | Chronic confusional state |
| E132.00 | Reactive confusion |
| E2A1000 | Mild memory disturbance |
| E2A1100 | Organic memory impairment |
| Eu04.00 | [X]Delirium, not induced by alcohol+other psychoactive subs |
| Eu04.11 | [X]Acute / subacute brain syndrome |
| Eu04.12 | [X]Acute / subacute confusional state, nonalcoholic |
| Eu04.13 | [X]Acute / subacute infective psychosis |
| Eu04.14 | [X]Acute / subacute organic reaction |
| Eu04.15 | [X]Acute / subacute psycho-organic reaction |
| Eu04000 | [X]Delirium not superimposed on dementia, so described |
| Eu04y00 | [X]Other delirium |
| Eu04y11 | [X]Delirium of mixed origin |
| Eu04z00 | [X]Delirium, unspecified |
| Eu05700 | [X]Mild cognitive disorder |
| Eu44y13 | [X]Psychogenic confusion |
| R009.00 | [D]Confusion |
| R009.11 | [D] Senile confusion |
| R009000 | [D]Toxic confusional state |
| R00z011 | [D]Memory deficit |
| R00zX00 | [D]Disorientation, unspecified |
| Ryu5.00 | [X]Symptoms/signs inv cognit, percept, emotion state & behav |
| Ryu5100 | [X]Oth & unspec symptom/sign involv cognit funct/awareness |
| Ryu5700 | [X]Disorientation, unspecified |
| Z7A1300 | Memory skills training |
| Z7A1500 | Memory retraining |
| Z7C1.00 | Impaired cognition |
| Z7C2200 | Unable to remember sounds |
| Z7CA100 | Isolated memory skills |
| Z7CE412 | Memory loss symptom |
| Z7CE414 | Memory disturbance |
| Z7CE415 | Loss of memory |
| Z7CE500 | Forgetful |
| Z7CE611 | Memory loss |
| Z7CE612 | Memory gone |
| Z7CE613 | Dysmnesia |
| Z7CE615 | Loss of memory |
| Z7CE616 | LOM - Loss of memory |
| Z7CEA00 | Impairment of registration |
| Z7CEA11 | Impairment of working memory |
| Z7CEA12 | Impairment of immediate recall |
| Z7CEA13 | Impairment of primary memory |
| Z7CEB12 | Poor memory for remote events |
| Z7CEC11 | Loss of memory for recent events |
| Z7CEC12 | No memory for recent events |
| Z7CEH00 | Memory impairment |
| Z7CEH11 | Memory dysfunction |
| Z7CEH12 | Memory deficit |
| Z7CEH13 | Bad memory |
| Z7CEH14 | Memory problem |
| Z7CEH15 | Poor memory |
| Z7CEI00 | Mixes past with present |
| Z7CEJ00 | Memory lapses |
| Z7CEK00 | Minor memory lapses |
| Z7CEL00 | Mild memory disturbance |
| Z7CEM00 | Distortion of memory |
| Z7CEN00 | Confabulation |
| Z7CEN11 | Invents experiences to compensate for loss of memory |
| Z7CEO00 | Momentary confabulation |
| Z7CEP00 | Fantastical confabulation |
| Z7CF200 | Has delayed recall |
| Z7CF800 | Poor short-term memory |
| Z7CF811 | Short-term memory loss |
| Z7CFA00 | Unable to recall random address at five minutes |
| Z7CFa00 | Unable to remember current year |
| Z7CFC00 | Unable to recall five digit number at five minutes |
| Z7CFe00 | Unable to remember name of current prime minister |
| Z7CFF00 | Forgets what was going to do |
| Z7CFg00 | Cannot remember names of intimates |
| Z7CFG00 | Forgets what was going to say |
| Z7CFh00 | Cannot remember birth dates of children |
| Z7CFH00 | Forgets recent activities |
| Z7CFi00 | Cannot remember wedding anniversary |
| Z7CFI00 | Forgets what has just done |
| Z7CFJ00 | Forgets what has just said |
| Z7CFK00 | Forgets what has just read |
| Z7CFL00 | Forgets what has just seen |
| Z7CFM00 | Forgets what just heard |
| Z7CFO00 | Poor long-term memory |
| Z7CFO11 | Long-term memory loss |
| Z7CFq00 | Unable to remember motor skills |
| Z7CFQ00 | Unable to remember own date of birth |
| Z7CFs00 | Unable to remember new motor skills |
| Z7CFS00 | Unable to remember own age |
| Z7CFS11 | Cannot remember own age |
| Z7CFU00 | Unable to remember day of the week |
| Z7CFW00 | Unable to remember today's date |
| Z7CFw00 | Memory aided by use of diary |
| Z7CFx00 | Memory aided by use of labels |
| Z7CFz00 | Memory aided by use of lists |
| Z7CGP00 | Delayed verbal memory |
| ZD11300 | Auditory memory therapy |
| ZS3..00 | Language-related cognitive disorder |

| **Codes for excluded pre-existing conditions** | | |
| --- | --- | --- |
| **Readcode** | **description** | **Condition** |
| A411.00 | Jakob-Cruetzfeldt disease | CJD |
| F11x700 | Cerebral degeneration due to Jakob-Creutzfeldt disease | CJD |
| Eu02100 | [X]Dementia in Creutzfeldt-Jakob disease | CJD |
| E02y100 | Drug-induced dementia | drug induced dementia |
| 43C3.11 | HIV positive | hiv |
| 43C4.00 | Human immunodeficiency virus test equivocal | hiv |
| 43d5.00 | HIV antibody/antigen (Duo) | hiv |
| 43h2.00 | HIV 1 PCR | hiv |
| 43h9.00 | HIV proviral deoxyribonucleic acid polymerase chain reaction | hiv |
| 43j7.00 | HIV 1 nucleic acid detection | hiv |
| 43w3.00 | Human immunodeficiency virus RNA/DNA ratio | hiv |
| 43W7.00 | HIV1 antibody level | hiv |
| 43W8.00 | HIV2 antibody level | hiv |
| 4J34.00 | HIV viral load | hiv |
| 4J35.00 | HIV p24 antigen level | hiv |
| 4J3F.00 | Human immunodeficiency virus viral load by log rank | hiv |
| 4JDT.00 | HIV serology | hiv |
| 4JDT000 | Rapid human immunodeficiency virus antibody test | hiv |
| 65QA.00 | AIDS carrier | hiv |
| 65VE.00 | Notification of AIDS | hiv |
| 66j..00 | Human immunodeficiency virus monitoring | hiv |
| 66j0.00 | Human immunodeficiency virus annual review | hiv |
| 9kl..00 | HIV pos gen health check serv declind - enhanc service admin | hiv |
| 9kl..11 | HIV positive general health check service declined | hiv |
| A788.00 | Acquired immune deficiency syndrome | hiv |
| A788.11 | Human immunodeficiency virus infection | hiv |
| A788000 | Acute human immunodeficiency virus infection | hiv |
| A788100 | Asymptomatic human immunodeficiency virus infection | hiv |
| A788200 | HIV infection with persistent generalised lymphadenopathy | hiv |
| A788300 | Human immunodeficiency virus with constitutional disease | hiv |
| A788400 | Human immunodeficiency virus with neurological disease | hiv |
| A788500 | Human immunodeficiency virus with secondary infection | hiv |
| A788600 | Human immunodeficiency virus with secondary cancers | hiv |
| A788U00 | HIV disease result/haematological+immunologic abnorms,NEC | hiv |
| A788V00 | HIV disease resulting in multiple diseases CE | hiv |
| A788W00 | HIV disease resulting in unspecified malignant neoplasm | hiv |
| A788X00 | HIV disease resulting/unspcf infectious+parasitic disease | hiv |
| A788y00 | Human immunodeficiency virus with other clinical findings | hiv |
| A788z00 | Acquired human immunodeficiency virus infection syndrome NOS | hiv |
| A789.00 | Human immunodef virus resulting in other disease | hiv |
| A789000 | HIV disease resulting in mycobacterial infection | hiv |
| A789100 | HIV disease resulting in cytomegaloviral disease | hiv |
| A789200 | HIV disease resulting in candidiasis | hiv |
| A789300 | HIV disease resulting in Pneumocystis carinii pneumonia | hiv |
| A789400 | HIV disease resulting in multiple infections | hiv |
| A789500 | HIV disease resulting in Kaposi's sarcoma | hiv |
| A789600 | HIV disease resulting in Burkitt's lymphoma | hiv |
| A789700 | HIV dis resulting oth types of non-Hodgkin's lymphoma | hiv |
| A789800 | HIV disease resulting in multiple malignant neoplasms | hiv |
| A789900 | HIV disease resulting in lymphoid interstitial pneumonitis | hiv |
| A789A00 | HIV disease resulting in wasting syndrome | hiv |
| A789X00 | HIV dis reslt/oth mal neopl/lymph,h'matopoetc+reltd tissu | hiv |
| AyuC.00 | [X]Human immunodeficiency virus disease | hiv |
| AyuC000 | [X]HIV disease resulting in other bacterial infections | hiv |
| AyuC100 | [X]HIV disease resulting in other viral infections | hiv |
| AyuC200 | [X]HIV disease resulting in other mycoses | hiv |
| AyuC300 | [X]HIV disease resulting in multiple infections | hiv |
| AyuC400 | [X]HIV disease resulting/other infectious+parasitic diseases | hiv |
| AyuC500 | [X]HIV disease resulting/unspcf infectious+parasitic disease | hiv |
| AyuC600 | [X]HIV disease resulting in other non-Hodgkin's lymphoma | hiv |
| AyuC700 | [X]HIV dis reslt/oth mal neopl/lymph,h'matopoetc+reltd tissu | hiv |
| AyuC800 | [X]HIV disease resulting in other malignant neoplasms | hiv |
| AyuC900 | [X]HIV disease resulting in unspecified malignant neoplasm | hiv |
| AyuCA00 | [X]HIV disease resulting in multiple diseases CE | hiv |
| AyuCB00 | [X]HIV disease result/haematological+immunologic abnorms,NEC | hiv |
| AyuCC00 | [X]HIV disease resulting in other specified conditions | hiv |
| AyuCD00 | [X]Unspecified human immunodeficiency virus [HIV] disease | hiv |
| R109.00 | [D]Laboratory evidence of human immunodefiency virus [HIV] | hiv |
| ZV01A00 | [V]Asymptomatic human immunodeficency virus infection status | hiv |
| Eu02400 | [X]Dementia in human immunodef virus [HIV] disease | hiv |
| F134.00 | Huntington's chorea | huntingdons |
| Eu02200 | [X]Dementia in Huntington's disease | huntingdons |
| 2987 | O/E -Parkinson flexion posture | parkinsons |
| 2987.11 | O/E - Parkinson posture | parkinsons |
| 2994 | O/E-festination-Parkinson gait | parkinsons |
| 2994.11 | O/E - Parkinson gait | parkinsons |
| 13Y5.00 | Parkinsons Society member | parkinsons |
| 147F.00 | History of Parkinson's disease | parkinsons |
| 297A.00 | O/E - Parkinsonian tremor | parkinsons |
| F11x900 | Cerebral degeneration in Parkinson's disease | parkinsons |
| F12..00 | Parkinson's disease | parkinsons |
| F120.00 | Paralysis agitans | parkinsons |
| F121.00 | Parkinsonism secondary to drugs | parkinsons |
| F121.11 | Drug induced parkinsonism | parkinsons |
| F122.00 | Malignant neuroleptic syndrome | parkinsons |
| F123.00 | Postencephalitic parkinsonism | parkinsons |
| F124.00 | Vascular parkinsonism | parkinsons |
| F12W.00 | Secondary parkinsonism due to other external agents | parkinsons |
| F12X.00 | Secondary parkinsonism, unspecified | parkinsons |
| F12z.00 | Parkinson's disease NOS | parkinsons |
| F130300 | Parkinsonism with orthostatic hypotension | parkinsons |
| Fyu2000 | [X]Other drug-induced secondary parkinsonism | parkinsons |
| Fyu2100 | [X]Other secondary parkinsonism | parkinsons |
| Fyu2200 | [X]Parkinsonism in diseases classified elsewhere | parkinsons |
| Fyu2900 | [X]Secondary parkinsonism, unspecified | parkinsons |
| Fyu2B00 | [X]Secondary parkinsonism due to other external agents | parkinsons |
| TJ6..00 | Adverse reaction to anticonvulsants/anti-parkinsonism drugs | parkinsons |
| TJ64.00 | Adverse reaction to antiparkinsonism drugs | parkinsons |
| TJ64z00 | Adverse reaction to antiparkinsonism drugs NOS | parkinsons |
| TJ6z.00 | Adverse reaction to anticonvulsant/antiparkinsonism drug NOS | parkinsons |
| U606.11 | [X] Adverse react to anticonvulsants & anti-parkinsons drugs | parkinsons |
| U606700 | [X]Antiparkinsonism drugs caus advers effects in therap use | parkinsons |
| U606711 | [X] Adverse reaction to antiparkinsonism drug | parkinsons |
| U606718 | [X] Adverse reaction to antiparkinsonism drugs NOS | parkinsons |
| U606719 | [X] Adverse react to anticonvuls or antiparkinson drug NOS | parkinsons |
| Eu02300 | [X]Dementia in Parkinson's disease | parkinsons |
| E012.00 | Other alcoholic dementia | alcohol dementia |
| E012.11 | Alcoholic dementia NOS | alcohol dementia |
| E012000 | Chronic alcoholic brain syndrome | alcohol dementia |
| Eu10711 | [X]Alcoholic dementia NOS | alcohol dementia |
| E041.00 | Dementia in conditions EC | dementia in other conditions |
| Eu02.00 | [X]Dementia in other diseases classified elsewhere | dementia in other conditions |
| Eu02y00 | [X]Dementia in other specified diseases classif elsewhere | dementia in other conditions |
| Eu02500 | [X]Lewy body dementia | lewy body |
| F116.00 | Lewy body disease | lewy body |
| Eu02000 | [X]Dementia in Pick's disease | Pick’s disease |
| F111.00 | Pick's disease | Pick’s disease |

| **Codes for dementia medicinal products** | | |
| --- | --- | --- |
| **Prodcode** | **Product name** | **Drug substance** |
| 9966 | Ebixa 5mg/0.5ml pump actuation oral solution (Lundbeck Ltd) | Memantine hydrochloride |
| 18800 | Ebixa 10mg tablets (Lundbeck Ltd) | Memantine hydrochloride |
| 5334 | Reminyl 12mg tablets (Shire Pharmaceuticals Ltd) | Galantamine hydrobromide |
| 53922 | Donepezil 10mg orodispersible tablets (Consilient Health Ltd) | Donepezil hydrochloride |
| 11546 | Exelon 1.5mg capsules (Novartis Pharmaceuticals UK Ltd) | Rivastigmine hydrogen tartrate |
| 5616 | Exelon 6mg capsules (Novartis Pharmaceuticals UK Ltd) | Rivastigmine hydrogen tartrate |
| 61385 | Nemdatine 10mg tablets (Actavis UK Ltd) | Memantine hydrochloride |
| 63226 | Prometax 9.5mg/24hours transdermal patches (Novartis Pharmaceuticals UK Ltd) | Rivastigmine |
| 61476 | Acumor XL 24mg capsules (Mylan) | Galantamine hydrobromide |
| 56600 | Donepezil 5mg tablets (Zentiva) | Donepezil hydrochloride |
| 24088 | Reminyl XL 24mg capsules (Shire Pharmaceuticals Ltd) | Galantamine hydrobromide |
| 58780 | Voleze 9.5mg/24hours transdermal patches (Focus Pharmaceuticals Ltd) | Rivastigmine |
| 68802 | Donepezil 5mg tablets (Waymade Healthcare Plc) | Donepezil hydrochloride |
| 48443 | Donepezil 10mg orodispersible tablets | Donepezil hydrochloride |
| 69564 | Prometax 4.6mg/24hours transdermal patches (Novartis Pharmaceuticals UK Ltd) | Rivastigmine |
| 9854 | Reminyl 4mg tablets (Shire Pharmaceuticals Ltd) | Galantamine hydrobromide |
| 55928 | Exelon 4.5mg capsules (Waymade Healthcare Plc) | Rivastigmine hydrogen tartrate |
| 18587 | Reminyl XL 8mg capsules (Shire Pharmaceuticals Ltd) | Galantamine hydrobromide |
| 37188 | Aricept Evess 10mg orodispersible tablets (Eisai Ltd) | Donepezil hydrochloride |
| 2931 | Donepezil 10mg tablets | Donepezil hydrochloride |
| 39363 | Ebixa 20mg tablets (Lundbeck Ltd) | Memantine hydrochloride |
| 62164 | Alzest 9.5mg/24hours transdermal patches (Dr Reddy's Laboratories (UK) Ltd) | Rivastigmine |
| 63217 | Donepezil 5mg tablets (A A H Pharmaceuticals Ltd) | Donepezil hydrochloride |
| 62925 | Acumor XL 16mg capsules (Mylan) | Galantamine hydrobromide |
| 63405 | Galsya XL 16mg capsules (Consilient Health Ltd) | Galantamine hydrobromide |
| 61920 | Luventa XL 8mg capsules (Fontus Health Ltd) | Galantamine hydrobromide |
| 62780 | Alzest 4.6mg/24hours transdermal patches (Dr Reddy's Laboratories (UK) Ltd) | Rivastigmine |
| 48482 | Galsya XL 8mg capsules (Consilient Health Ltd) | Galantamine hydrobromide |
| 59330 | Voleze 4.6mg/24hours transdermal patches (Focus Pharmaceuticals Ltd) | Rivastigmine |
| 55720 | Gatalin XL 24mg capsules (Aspire Pharma Ltd) | Galantamine hydrobromide |
| 37957 | Exelon 9.5mg/24hours transdermal patches (Novartis Pharmaceuticals UK Ltd) | Rivastigmine |
| 57139 | Ebixa 10mg tablets (DE Pharmaceuticals) | Memantine hydrochloride |
| 61618 | Nemdatine 20mg tablets (Actavis UK Ltd) | Memantine hydrochloride |
| 62867 | Gazylan XL 16mg capsules (Teva UK Ltd) | Galantamine hydrobromide |
| 65534 | Donepezil 5mg orodispersible tablets sugar free (A A H Pharmaceuticals Ltd) | Donepezil hydrochloride |
| 35088 | Donepezil 10mg orodispersible tablets sugar free | Donepezil hydrochloride |
| 61921 | Luventa XL 24mg capsules (Fontus Health Ltd) | Galantamine hydrobromide |
| 37444 | Exelon 4.6mg/24hours transdermal patches (Novartis Pharmaceuticals UK Ltd) | Rivastigmine |
| 62868 | Gazylan XL 24mg capsules (Teva UK Ltd) | Galantamine hydrobromide |
| 60107 | Donepezil 5mg tablets (Alliance Healthcare (Distribution) Ltd) | Donepezil hydrochloride |
| 20140 | Reminyl XL 16mg capsules (Shire Pharmaceuticals Ltd) | Galantamine hydrobromide |
| 65573 | Gazylan XL 8mg capsules (Teva UK Ltd) | Galantamine hydrobromide |
| 53842 | Aricept 5mg tablets (Waymade Healthcare Plc) | Donepezil hydrochloride |
| 11716 | Exelon 3mg capsules (Novartis Pharmaceuticals UK Ltd) | Rivastigmine hydrogen tartrate |
| 63360 | Luventa XL 16mg capsules (Fontus Health Ltd) | Galantamine hydrobromide |
| 58937 | Exelon 13.3mg/24hours transdermal patches (Novartis Pharmaceuticals UK Ltd) | Rivastigmine |
| 58709 | Donepezil 10mg tablets (A A H Pharmaceuticals Ltd) | Donepezil hydrochloride |
| 36848 | Aricept Evess 5mg orodispersible tablets (Eisai Ltd) | Donepezil hydrochloride |
| 56709 | Gatalin XL 16mg capsules (Aspire Pharma Ltd) | Galantamine hydrobromide |
| 67593 | Donepezil 10mg tablets (Zentiva) | Donepezil hydrochloride |
| 59871 | Donepezil 10mg/5ml oral suspension | Donepezil hydrochloride |
| 2930 | Donepezil 5mg tablets | Donepezil hydrochloride |
| 35179 | Donepezil 5mg orodispersible tablets sugar free | Donepezil hydrochloride |
| 56421 | Gatalin XL 8mg capsules (Aspire Pharma Ltd) | Galantamine hydrobromide |
| 68493 | Nemdatine tablets treatment initiation pack (Actavis UK Ltd) |  |
| 61676 | Donepezil 1mg/ml oral solution sugar free | Donepezil hydrochloride |
| 29288 | Reminyl 4mg/ml oral solution (Shire Pharmaceuticals Ltd) | Galantamine hydrobromide |
| 5247 | Aricept 10mg tablets (Eisai Ltd) | Donepezil hydrochloride |
| 5400 | Aricept 5mg tablets (Eisai Ltd) | Donepezil hydrochloride |
| 58947 | Donepezil 10mg tablets (Accord Healthcare Ltd) | Donepezil hydrochloride |
| 39362 | Ebixa tablets treatment initiation pack (Lundbeck Ltd) | Memantine Hydrochloride |
| 48015 | Galsya XL 24mg capsules (Consilient Health Ltd) | Galantamine hydrobromide |
| 18062 | Reminyl 8mg tablets (Shire Pharmaceuticals Ltd) | Galantamine hydrobromide |
| 20404 | Exelon 4.5mg capsules (Novartis Pharmaceuticals UK Ltd) | Rivastigmine hydrogen tartrate |
| 18556 | Exelon 2mg/ml oral solution (Novartis Pharmaceuticals UK Ltd) | Rivastigmine hydrogen tartrate |
| 48442 | Donepezil 5mg orodispersible tablets | Donepezil hydrochloride |
