# Supplementary material for: Identifying individuals at high risk for dementia in primary care: Development and validation of the DemRisk risk prediction model using routinely collected patient data
Source: PLoS One. 2024 Oct 4;19(10):e0310712. doi: 10.1371/journal.pone.0310712 (PMC11452046; doi:10.1371/journal.pone.0310712)
Supplement: S1 Checklist — (DOCX) [file pone.0310712.s003.docx]

STROBE Statement—checklist of items that should be included in reports of observational studies

|  | Item No. | Recommendation | Page  No. | Relevant text from manuscript |
| --- | --- | --- | --- | --- |
| **Title and abstract** | 1 | (*a*) Indicate the study’s design with a commonly used term in the title or the abstract | 2 | “Risk prediction algorithms were developed and validated using multivariable Cox regression” |
|  |  | (*b*) Provide in the abstract an informative and balanced summary of what was done and what was found | 2-3 |  |
| Introduction | | | |  |
| Background/rationale | 2 | Explain the scientific background and rationale for the investigation being reported | 3-5 |  |
| Objectives | 3 | State specific objectives, including any prespecified hypotheses | 5 | Our study aims… |
| Methods | | | |  |
| Study design | 4 | Present key elements of study design early in the paper | 6-10 |  |
| Setting | 5 | Describe the setting, locations, and relevant dates, including periods of recruitment, exposure, follow-up, and data collection | 6-10 |  |
| Participants | 6 | (*a*) *Cohort study*—Give the eligibility criteria, and the sources and methods of selection of participants. Describe methods of follow-up  *Case-control study*—Give the eligibility criteria, and the sources and methods of case ascertainment and control selection. Give the rationale for the choice of cases and controls  *Cross-sectional study*—Give the eligibility criteria, and the sources and methods of selection of participants | 7-8 |  |
|  |  | (*b*) *Cohort study*—For matched studies, give matching criteria and number of exposed and unexposed  *Case-control study*—For matched studies, give matching criteria and the number of controls per case | NA |  |
| Variables | 7 | Clearly define all outcomes, exposures, predictors, potential confounders, and effect modifiers. Give diagnostic criteria, if applicable | 8-10 |  |
| Data sources/ measurement | 8* | For each variable of interest, give sources of data and details of methods of assessment (measurement). Describe comparability of assessment methods if there is more than one group | *6-7, 8-10* |  |
| Bias | 9 | Describe any efforts to address potential sources of bias | 6,  8, | GOLD was linked to two additional datasets….  This produced a cohort with an age distribution broadly representative… |
| Study size | 10 | Explain how the study size was arrived at | 10 |  |

Continued on next page

| Quantitative variables | 11 | Explain how quantitative variables were handled in the analyses. If applicable, describe which groupings were chosen and why | 9-10 |  |
| --- | --- | --- | --- | --- |
| Statistical methods | 12 | (*a*) Describe all statistical methods, including those used to control for confounding | 10-13 |  |
|  |  | (*b*) Describe any methods used to examine subgroups and interactions | 12  16 | To assess heterogeneity…  “We explored…interaction between…” |
|  |  | (*c*) Explain how missing data were addressed | 11, 29, S1 File | Passage on missing data |
|  |  | (*d*) *Cohort study*—If applicable, explain how loss to follow-up was addressed  *Case-control study*—If applicable, explain how matching of cases and controls was addressed  *Cross-sectional study*—If applicable, describe analytical methods taking account of sampling strategy | 13 | Passage on risk classification |
|  |  | (*e*) Describe any sensitivity analyses |  |  |
| Results | | | | |
| Participants | 13* | (a) Report numbers of individuals at each stage of study—eg numbers potentially eligible, examined for eligibility, confirmed eligible, included in the study, completing follow-up, and analysed | 14, Table 1  22, Table A2 |  |
|  |  | (b) Give reasons for non-participation at each stage | 8 | Figure 1 |
|  |  | (c) Consider use of a flow diagram | 8 | Figure 1 |
| Descriptive data | 14* | (a) Give characteristics of study participants (eg demographic, clinical, social) and information on exposures and potential confounders | 14, Table 1  22, Table A2 |  |
|  |  | (b) Indicate number of participants with missing data for each variable of interest | 11 | Passage on missing data |
|  |  | (c) *Cohort study*—Summarise follow-up time (eg, average and total amount) | 14, Table 1  22, Table A2 |  |
| Outcome data | 15* | *Cohort study*—Report numbers of outcome events or summary measures over time | 14, Table 1  22, Table A2 |  |
|  |  | *Case-control study—*Report numbers in each exposure category, or summary measures of exposure | *NA* |  |
|  |  | *Cross-sectional study—*Report numbers of outcome events or summary measures | *NA* |  |
| Main results | 16 | (*a*) Give unadjusted estimates and, if applicable, confounder-adjusted estimates and their precision (eg, 95% confidence interval). Make clear which confounders were adjusted for and why they were included | 16, Table 2  19, Table 4  22, Table A3  23, Table 6 |  |
|  |  | (*b*) Report category boundaries when continuous variables were categorized | Table 1 | Practice IMD |
|  |  | (*c*) If relevant, consider translating estimates of relative risk into absolute risk for a meaningful time period | 21, text and Table 5  24, text and Table 7 | Reports estimates of true positives and false negatives for 5-year follow-up at various risk thresholds |

Continued on next page

| Other analyses | 17 | Report other analyses done—eg analyses of subgroups and interactions, and sensitivity analyses | P21  P22  P25 | Practice-level C-statistics  Comparison to DRS and age-only models  Comparison to DRS and age-only models |
| --- | --- | --- | --- | --- |
| Discussion | | | | |
| Key results | 18 | Summarise key results with reference to study objectives | 25 | Discussion section |
| Limitations | 19 | Discuss limitations of the study, taking into account sources of potential bias or imprecision. Discuss both direction and magnitude of any potential bias | 27-29 | Passage on strengths and limitations |
| Interpretation | 20 | Give a cautious overall interpretation of results considering objectives, limitations, multiplicity of analyses, results from similar studies, and other relevant evidence | 31-32 | Passage on implications and conclusions |
| Generalisability | 21 | Discuss the generalisability (external validity) of the study results | 31 | Passage on implications |
| Other information | |  | | |
| Funding | 22 | Give the source of funding and the role of the funders for the present study and, if applicable, for the original study on which the present article is based |  | Separate funding statement |

*Give information separately for cases and controls in case-control studies and, if applicable, for exposed and unexposed groups in cohort and cross-sectional studies.

**Note:** An Explanation and Elaboration article discusses each checklist item and gives methodological background and published examples of transparent reporting. The STROBE checklist is best used in conjunction with this article (freely available on the Web sites of PLoS Medicine at http://www.plosmedicine.org/, Annals of Internal Medicine at http://www.annals.org/, and Epidemiology at http://www.epidem.com/). Information on the STROBE Initiative is available at www.strobe-statement.org.
