# Supplementary material for: Identifying individuals at high risk for dementia in primary care: Development and validation of the DemRisk risk prediction model using routinely collected patient data
Source: PLoS One. 2024 Oct 4;19(10):e0310712. doi: 10.1371/journal.pone.0310712 (PMC11452046; doi:10.1371/journal.pone.0310712)
Supplement: S2 Checklist — (DOCX) [file pone.0310712.s004.docx]

**TRIPOD-Cluster checklist of items to include when reporting a study developing or validating a multivariable prediction model using clustered data**

| **#** | **Description** | **Page #** |
| --- | --- | --- |
| **Title and abstract** | |  |
| 1 | Identify the study as developing and/or validating a multivariable prediction model, the target population, and the outcome to be predicted. | 2 |
| 2 | Provide a summary of research objectives, setting, participants, data source, sample size, predictors,  outcome, statistical analysis, results, and conclusions.* | 2 |
| **Introduction** | |  |
| 3a | Explain the medical context (including whether diagnostic or prognostic) and rationale for developing or  validating the prediction model, including references to existing models, and the advantages of the study design.* | 3-5 |
| 3b | Specify the objectives, including whether the study describes the development or validation of the  model.* | 5 |
| **Methods** | |  |
| 4a | Describe eligibility criteria for participants and datasets.* | 6-8 |
| 4b | Describe the origin of the data, and how the data were identified, requested, and collected. | 6 |
| 5 | Explain how the sample size was arrived at.* | 10 |
| 6a | Define the outcome that is predicted by the model, including how and when assessed.* | 8 |
| 6b | Define all predictors used in developing or validating the model, including how and when measured.* | 9-10 |
| 7a | Describe how the data were prepared for analysis, including any cleaning, harmonisation, linkage, and  quality checks. | 6-7 |
| 7b | Describe the method for assessing risk of bias and applicability in the individual clusters (eg, using PROBAST). |  |
| 7c | For validation, identify any differences in definition and measurement from the development data (eg,  setting, eligibility criteria, outcome, predictors).* | NA |
| 7d | Describe how missing data were handled.* | 11, 29, S1 File |
| 8a | Describe how predictors were handled in the analyses. | 9-10 |
| 8b | Specify the type of model, all model-building procedures (eg, any predictor selection and penalisation), and method for validation.* | 11-12 |
| 8c | Describe how any heterogeneity across clusters (eg, studies or settings) in model parameter values was  handled. | 12 |
| 8d | For validation, describe how the predictions were calculated. | 13, S1 File |
| 8e | Specify all measures used to assess model performance (eg, calibration, discrimination, and decision  curve analysis) and, if relevant, to compare multiple models. | 11-12 |
| 8f | Describe how any heterogeneity across clusters (eg, studies or settings) in model performance was  handled and quantified. | 12 |
| 8g | Describe any model updating (eg, recalibration) arising from the validation, either overall or for particular populations or settings.* | NA |
| 9 | Describe any planned subgroup or sensitivity analysis, (eg, assessing performance according to sources of  bias, participant characteristics, setting). | 12 |
| **Results** | |  |
| 10a | Describe the number of clusters and participants from data identified through to data analysed. A flow  chart may be helpful.* | Fig 1 |
| 10b | Report the characteristics overall and where applicable for each data source or setting, including the key dates, predictors, treatments received, sample size, number of outcome events, follow-up time, and  amount of missing data.* | Table 1; Table A2 in S1 File |
| 10c | For validation, show a comparison with the development data of the distribution of important variables  (demographics, predictors, and outcome). | Table 1; Table A2 in S1 File |
| 11 | Report the results of the risk of bias assessment in the individual clusters. |  |
| 12a | Report the results of any across-cluster heterogeneity assessments that led to subsequent actions during  the model’s development (eg, inclusion or exclusion of particular predictors or clusters). | NA |
| 12b | Present the final prediction model (ie, all regression coefficients, and model intercept or baseline estimate  of the outcome at a given time point) and explain how to use it for predictions in new individuals.* | Table 4; Table 6; S1 File |
| 13a | Report performance measures (with uncertainty intervals) for the prediction model, overall and for each  cluster. | Table 3; Table A4 in S1 file |

| 13b | Report results of any heterogeneity across clusters in model performance. | 21 |
| --- | --- | --- |
| 14 | Report the results from any model updating (including the updated model equation and subsequent  performance), overall and for each cluster.* | NA |
| 15 | Report results from any subgroup or sensitivity analysis. |  |
| **Discussion** | |  |
| 16a | Give an overall interpretation of the main results, including heterogeneity across clusters in model performance, in the context of the objectives and previous studies.* | 25-27 |
| 16b | For validation, discuss the results with reference to the model performance in the development data, and  in any previous validations. |  |
| 16c | Discuss the strengths of the study and any limitations (eg, missing or incomplete data, non-  representativeness, data harmonisation problems).* | 27-29 |
| 17 | Discuss the potential use of the model and implications for future research, with specific view to generalisability and applicability of the model across different settings or (sub)populations.* | 31 |
| **Other information** | |  |
| 18 | Provide information about the availability of supplementary resources (eg, study protocol, analysis code, datasets).* | S1 file; S2 file |
| 19 | Give the source of funding and the role of the funders for the present study. | Funding statement |

This checklist is taken from Debray TPA, Collins GS, Riley RD et al. Transparent reporting of multivariable prediction models developed or validated using clustered data: TRIPOD-Cluster checklist. *BMJ* 2022;378:e071018; doi:10.1136/bmj- 2022-071018.

PROBAST=prediction model risk-of-bias assessment tool.

*****Item text is an adaptation of one or more existing items from the original TRIPOD (transparent reporting of a multivariable prediction model for individual prognosis or diagnosis) checklist.
