# Supplementary material for: Identifying individuals at high risk for dementia in primary care: Development and validation of the DemRisk risk prediction model using routinely collected patient data
Source: PLoS One. 2024 Oct 4;19(10):e0310712. doi: 10.1371/journal.pone.0310712 (PMC11452046; doi:10.1371/journal.pone.0310712)
Supplement: Attachment — Submitted filename: Response to Reviewers.docx [file pone.0310712.s005.docx]

**RE: Identifying individuals at high risk for dementia in primary care: development and validation of the DemRisk risk prediction model using routinely collected patient data**

Please find our point-by-point response to the Editor and reviewer’s comments below

Journal Requirements:

1. When submitting your revision, we need you to address these additional requirements.

Please ensure that your manuscript meets PLOS ONE's style requirements, including those for file naming. The PLOS ONE style templates can be found at 
[https://journals.plos.org/plosone/s/file?id=wjVg/PLOSOne_formatting_sample_main_body.pdf [journals.plos.org]](https://urldefense.com/v3/__https:/journals.plos.org/plosone/s/file?id=wjVg*PLOSOne_formatting_sample_main_body.pdf__;Lw!!PDiH4ENfjr2_Jw!FIVVin6AzbuCQCI_xXQ2rUNT6TucFeOt1J4fkQgedfcHPt4G6cC5LGUxczOXjt0UarTgATBaCtoBSHF8Us7o6tLqgg$) and 
[https://journals.plos.org/plosone/s/file?id=ba62/PLOSOne_formatting_sample_title_authors_affiliations.pdf [journals.plos.org]](https://urldefense.com/v3/__https:/journals.plos.org/plosone/s/file?id=ba62*PLOSOne_formatting_sample_title_authors_affiliations.pdf__;Lw!!PDiH4ENfjr2_Jw!FIVVin6AzbuCQCI_xXQ2rUNT6TucFeOt1J4fkQgedfcHPt4G6cC5LGUxczOXjt0UarTgATBaCtoBSHF8Us45aADMKQ$)

*We have done our best to ensure that our manuscript meets the journal’s style requirements, including file naming.*

2. Please note that PLOS ONE has specific guidelines on code sharing for submissions in which author-generated code underpins the findings in the manuscript. In these cases, we expect all author-generated code to be made available without restrictions upon publication of the work. Please review our guidelines at [https://journals.plos.org/plosone/s/materials-and-software-sharing#loc-sharing-code [journals.plos.org]](https://urldefense.com/v3/__https:/journals.plos.org/plosone/s/materials-and-software-sharing*loc-sharing-code__;Iw!!PDiH4ENfjr2_Jw!FIVVin6AzbuCQCI_xXQ2rUNT6TucFeOt1J4fkQgedfcHPt4G6cC5LGUxczOXjt0UarTgATBaCtoBSHF8Us6YSeys8g$) and ensure that your code is shared in a way that follows best practice and facilitates reproducibility and reuse.

*We have posted all of the Read code-lists required to construct the variables used in the study from the CPRD dataset onto the GitHub public depository and provided the URL for this in our Data Availability statement, as copied below:*

*“The data used in this study was obtained via the Clinical Practice Research Datalink (CPRD). CPRD provides a research service which provides representative, longitudinal real-time anonymized patient electronic health records data from primary care and other health services across the UK. The licensing agreement between University of Manchester and CPRD, and the data governance of CPRD prevent the sharing or distribution of patient data to other individuals. Hence any requests for access to data from the study should be addressed to cprdenquiries@mhra.gov.uk. All researchers requiring access will require approval of their proposals from CPRD before data release.*

*The Read code-lists used to generate all the variables used in the study from the CPRD GOLD dataset have been made publicly available at:* [*https://github.com/C5thyM/demrisk*](https://github.com/C5thyM/demrisk)*.”*

3. Your ethics statement should only appear in the Methods section of your manuscript. If your ethics statement is written in any section besides the Methods, please move it to the Methods section and delete it from any other section. Please ensure that your ethics statement is included in your manuscript, as the ethics statement entered into the online submission form will not be published alongside your manuscript.

*We have added the ethics statement to the Methods section:*

*“Access to CPRD GOLD was obtained under licence from the UK Medicines and Healthcare products Regulatory Agency. CPRD GOLD consists of routinely collected data that has been pseudonymised for the purposes of research, for which informed consent was not required. The study was approved by the independent scientific advisory committee for Clinical Practice Research Datalink research (protocol No 18_163R).”*

[Note: HTML markup is below. Please do not edit.]

Reviewers' comments:

Reviewer's Responses to Questions

**Comments to the Author**

5. Review Comments to the Author

**Reviewer #1: Line 215 - it would be beneficial if the time period for polypharmacy and pulse pressure are given in the text (not just tables).**

*We have revised the text to include this information, which – due to other changes – now appears on lines 219 and 226.*

**Line 225 - there are grave statistical consequences of using a missing data category instead of imputing. Missing data indicator is no longer encouraged when analysing data It is possible to see that the amount of missing data for the health indicators is not high, and most older people will have these data collected regularly due to them having at least one QoF condition. The Walters, Hardoon paper showed that it was possible to impute these data adequately. For smoking status, those that are missing are most likely to be non-smokers** [https://bmjopen.bmj.com/content/bmjopen/4/4/e004958.full.pdf [bmjopen.bmj.com]](https://urldefense.com/v3/__https:/bmjopen.bmj.com/content/bmjopen/4/4/e004958.full.pdf__;!!PDiH4ENfjr2_Jw!FIVVin6AzbuCQCI_xXQ2rUNT6TucFeOt1J4fkQgedfcHPt4G6cC5LGUxczOXjt0UarTgATBaCtoBSHF8Us5AYixGhQ$)

*The debate around the use of the Missing Value Method (MVM) is not as clear cut as the reviewer suggests (see below). Nonetheless, we re-visited our decision and ran an analysis using multiple imputation (MI) (fully conditional specification with 10 imputed datasets) for comparison purposes. We discovered that the two methods produced identical C-statistics (0.781 for the 60-79 cohort) and Royston Ds (1.74), plus risk factor coefficients that in almost every case were near-identical. This indicated to us that the missingness was non-informative (see below). On this basis we decided to change the manuscript to report the results obtained using MI, since the models are slightly simpler without the missing value indicators and the method has greater “face validity”.*

*However, this means that were our models to be deployed in practice, a separate method would be required to derive risk scores for cases with missing values (for various reasons MI itself cannot be deployed, not least because the Fully Conditional Specification method requires knowledge of the future outcome). To address this we now include a passage in the S1 File demonstrating a simple mean imputation method that produces reasonably accurate risk scores for more than 99% of these cases.*

*For context: the main criticism of MVM is that it can produce biased estimates of the co-effs for some risk factors. However, other authors point out that while MI will produce more accurate coefficients when the assumption that data is missing at random is correct, MI itself will be biased if missingness is informative. Recently, Ness et al (*[*https://doi.org/10.1145/3580305.3599911*](https://doi.org/10.1145/3580305.3599911)*) demonstrated both theoretically and empirically that in the presence of informative missingness MVM has greater predictive performance compared to imputation methods, and when missingness is not informative the inclusion of missing value indicators does not negatively affect performance. They recommend that when predictive performance is prioritised over precise imputations “MVM plus mean imputation is a strong method while being much more efficient than more complicated imputation schemes.” We mention this only to demonstrate that we had a good rationale for our original analysis decision in this respect.*

*Having now adopted the MI method, missing data on smoking status has been subjected to multiple imputation, hence there is no necessity to collapse this category with another.*

**Line 253 - the outcome is time to dementia diagnosis**

*We have revised the first sentence of the paragraph to read “We applied Cox proportional hazards regression models to build our predictive models, using time to dementia diagnosis as the outcome.” (new Line 259)*

**In the participants section, you say you exclude those with less than one year follow-up, but the lower quartile follow-up is 0.96, so assume these and the 25% with shorter follow-up got dementia in the first year of follow-up?**

*We now realise that our text was in error. We did not exclude patients with less than one year of follow-up; rather, we excluded patients with less than one full year of EHR data prior to their date of study entry. The reviewer is correct in saying that 25% had less than one year of follow-up, but not all of these got dementia in the first year of follow-up: some died or moved away from the practice, or the practice may have ceased contributing to CPRD. However, all patients had a minimum of one full year of data prior to the date of study entry (ie the start of follow-up). We have revised the text to read “We excluded patients with a code for dementia recorded prior to study entry, with less than one year of continuous registration in the practice prior to study entry, or with less than one full year of consultation data prior to study entry.” (new line 170)*

**Table 1 - sight impairment must be under-recorded as many people of that age would wear glasses for some degree of sight impairment, but many will not consult their GP for conditions that can be helped by opticians.**

*Many conditions are under-recorded in the EHR. This is an acknowledged limitation of the data and may be one reason why some factors do not appear in the algorithms. However, we do expect most of the more severe cases of sight impairment to be recorded, where for example opticians have been unable to help or have advised a person to consult their GP, as they are advised to do in the UK.*

**Table 1 - define GP consultations involving a 3rd party (it only becomes when reading univariable associations text).**

***We have revised the text in the table to read “Number of GP consultations involving third party (e.g. relative/friend) in last year”.*

Table 1 - define anticholinergic burden.**

*We have added a definition and related reference to the main text at line 213, in the passage about predictive factors: “We also included measures of polypharmacy (number of different prescribed medicines over previous 12 months) and of anticholinergic burden (by which each drug is assigned a score of 1, 2 or 3 depending upon degree of anticholinergic effect and the scores totalled.(33)”*

**Table 2 - no need to put ns in the table. It is better to allow readers to make their own minds up from the estimate and 95% CI.**

*We have removed the ns from table 2. We have also removed them from the corresponding table (A5) in the S1 File for the older patient cohort*

**Line 364 - "The two cohorts..." this sentence sounds as if it has not been checked. If it has not been checked then remove (or check it). If it has been checked then modify the sentence to make it clear.**

*For clarity we have revised the sentence to read “The validation and development cohorts….” (new line 381)*
 **Line 398 (and beginning of the paragraph) - I think total number of participants or person years is wrong as they read as the same.**

*We thank the reviewer for spotting this mistake. We have corrected the number of participants to be 175,131* **Line 466 - Higher blood pressure in midlife is associated with higher dementia risk. Blood pressure decreases in later life (usually) so lower blood pressure measured in later life as here is a risk factor for dementia. See Lancet Commission for Dementia, 2020.**

*We have revised the text to include this point: “These associations are all in line with existing evidence,(46-49) though it is worth noting that obesity and hypertension measured in midlife predict future dementia and it is thought that weight loss and lower blood pressure in later life may potentially be symptoms of developing dementia.(10)” (new line 501)*

**Reviewer #2:** Comments to Authors:

**The aims of the current study were to develop risk models with improved ability to identify patients at higher risk of future dementia from their UK primary care record, by investigating a wider set of potential predictive factors, constructing development and validation cohorts more representative of the target population, and by increasing the completeness of information on dementia diagnosis using linked secondary care data. This is a robust study, and I read with great interest. However, I have a few concerns.

- Major points
1. Generally, patients who visit GPs are at higher risk of future dementia or may have great interest in preventing dementia. Namely, the authors’ study may have selection bias. They may be moderate or severe dementia cases. Mild cases may not be recorded in the GPs EHRs.**

*The GOLD dataset includes data on all patients registered with participating GPs - together with data on all their consultations at the GP practice for any reason - and almost all adults in the UK are registered with a GP practice. Our cohorts included all registered patients who at baseline had no symptoms of dementia or prodromal dementia (eg memory loss codes); there was no selection on the basis of the future outcome. Thus our sample includes all patients who went on to develop mild dementia as well as more severe forms.*

*However, it is very likely that mild cases were less likely to be classed as positive cases for the purpose of model building.* *There is no easy or reliable way to identify mild dementia through use of Read codes: many of the related symptoms such as mild memory loss are shared by many other conditions, even just normal ageing. For this reason positive cases were defined (as they have been in other EHR-based studies) as those with a future diagnosis that specifically stated dementia, or prescription of a dementia-specific drug. We have added some relevant text to the strengths and limitations section, at line 552:*

*“Diagnosis usually occurs during the mid-stage of dementia, typically two-to-four years after first onset,(54) and our models are focused on picking up these individuals before they show clear symptoms. Early-stage dementia cannot be reliably identified through use of Read codes, as the key symptoms such as mild memory loss are common to many other conditions, including normal ageing. Identification of patients at risk of early-stage dementia may require a different form of approach.”*

**2. According to IQR of follow-ups, about a quarter of patients for development cohort 60-79 years (0.96, 5) and validation cohort 60-79 years (0.99, 5) were lost to follow-up before a year (Table 1). Characteristics of early censored participants could be different from participants who followed up 5 years. Some of them might develop dementia when followed up longer.**

*We agree. Early censored participants are more likely to be older and more ill and to have died in the first year of follow-up. Many patients who were censored due to leaving their practice, say, could have developed dementia at a later date. It is important to note that the Cox analysis does not assume that censored patients never developed dementia: these patients are assumed to come from the same population as patients with similar characteristics who remained with their practice. This is one of the main advantages of the Cox model, in that it models length of survival (in this case, time to dementia diagnosis) taking account of such covariates.*

**3. Prescribed medications could have overlapped with some medical conditions (ever recorded). When both sets of variables are entered in the same model, multicollinearity is expected. Usually, working operational definitions are developed by combining records of medication and medical conditions.**

*We coded medications as different factors from medical conditions on the recommendation of Prof Darren Ashcroft, Professor of Pharmacoepidemiology and a member of the research team with long experience of analysing CPRD. Many medications are used for a variety of conditions and not restricted to a single one. In addition, many have biological effects that are distinct from (and can be opposite to) the effects of the medical condition itself. Our paper includes a good example in the case of depression and related medications. Diagnosed depression was a weak predictor of future dementia, whereas use of SSRIs and anti-depressive drugs other than TCAs were strong predictors. TCAs themselves were negatively related, which may be related to their anti-inflammatory properties. Notably, when we explored combining depression diagnosis and medications into a single variable fit indices substantially decreased, supporting their use as separate factors.*

*Multicollinearity was an issue that we took into account during the stepwise reduction procedure in deciding between factors of similar strength for removal. We have revised the text at line 277 to read “The reduction was curated in that decisions about factors to be dropped or retained at each step were based not only on statistical considerations (p-values, hazard ratios and multicollinearity), but also on clinical and practical considerations to ensure that the reduced model made clinical sense…”*

*We also now report on line 370 for the 60-79 model that “Excepting the correlation between age and age-squared, each factor in the final model had a variance inflation factor (VIF) of <2.0, generally considered to be low multicollinearity.(45)”. Likewise at line 441 for the 80plus model.*

**4. Although CPRD has unreliable information such as education for older patients, education is a major factor in elucidating the relationship with dementia. Is there any proxy measure that can be used instead of education?**

*We know of no measure in CPRD that would make a suitable proxy. There are occupation codes but we know that those are rarely completed and most patients in our population will be retired. Also, the link between occupation and education may be only weak. However, education is correlated with practice IMD, age and BMI all of which are part of our model and hence capture some of the relationship with education.*

**5. IMD represents geographic communities. The authors may consider additionally applying multilevel Cox proportional hazards regression models (line 254, 259).**

*We are aware that the dataset is two-level, though our models have been fitted as single-level. We have now fitted two-level Cox regressions to our final models for each age-cohort. In both cases the indices of predictive performance were identical or very similar to those obtained using single-level models.*

*We have presented this in the manuscript as a sensitive analysis at lines 284, 391 and 456. We considered redoing all of our analyses using multi-level models but decided that the analysis time required to do this would be far too excessive. The two sensitivity Cox regressions we now include took around 50 and 30 hours to run respectively, having switched to a multiple imputation approach. Allowing for the MI, the study as a whole requires a minimum of 200 univariate and 40 multivariate Cox regressions, which would amount to several weeks of analysis time. In additional, multi-level models often fail to converge and it would be highly problematic if that occurred for a proportion of the analyses.*

**6. With 60 evidence-based risk factors, the DemRisk models might be overfitted. This may lead to lack of generalizability. Models should be parsimonious. I suggest Chapter 7, Algorithms to Live By: The Computer Science of Human Decisions, written by Brian Christian and Tom Griffiths.”**

*The Analysis section of our paper includes a passage on “Adequacy of sample size”, where we used the methods of Riley et al to determine the maximum number of factors we could evaluate whilst retaining an acceptably small degree of overfitting (and other forms of bias). The numbers reported there are 200 factors for the younger cohort and 100 for the (smaller) older cohort. 60 factors is well within this range. In addition, all of our risk factors were prespecified with a good evidence base and were only included in our final models if the direction of effect concurred with the evidence. We have revised the manuscript text at line 522 to now read:*

*“We investigated a pool of 60 potential factors and our final models include up to 19 predictors. Large predictive models are at higher risk of overfitting. However, we think DemRisk unlikely to be overfitted. First, all of our factors were prespecified on the basis of possessing a good evidence base and only included in our final models if their direction of effect was in line with the literature; this limits the potential for overfitting through data-driven variable selection.(53) Second, the great majority of the included factors also feature in more than one previously published dementia prediction model. Third, our sample size analysis indicated that we had sufficient data to build models with up to 200 factors whilst keeping overfitting and other biases within acceptable limits.”*

*We used stepwise removal to make our models parsimonious and our final algorithms included at most 19 factors. Efforts to reduce the models further resulted in notable reductions in predictive performance. Since we had more than enough cases to support estimating 19 (or more) factors (see previous paragraph) we opted for this model in order to preserve predictive performance. We note that more parsimonious models for dementia exist but these have had only low predictive ability. The 2024 report from the Lancet Commission on Dementia identifies 14 factors associated with the risk of incident dementia, also illustrating the multi-faceted nature of the condition.*

*Nevertheless, the crucial test for the generalisability of a model is how it performs in external validation datasets. This point is made in the Discussion section. We have now revised the passage there to give increased emphasis: “Further research is needed to more firmly establish the external validity of the models. Although our sample sizes were large relative to the number of factors investigated and all the factors were evidence-based, our development and validation cohorts were both drawn from the same population of English GP practices using the Vision patient record system only. Validation work is strongly advised in relation to other countries in the UK and other record systems, such as the widely used EMIS and SystmOne….”*

**- Minor points
1. The authors described their data source in detail so that readers can easily understand CPRD GOLD. Although this is a novel study, the authors may summarize a bit more (line 134-141, 142-149, 150-157).**

*We have endeavoured to reduce the length of this section somewhat.*

**2. Compared to the long list of study strengths and their implications, the discussion provided in the study is somewhat short. The explanation may not provide strong evidence for the authors’ large prediction models. Would you please add more evidence by reviewing other previous studies?**

*We have added a new section after Strengths and Limitations, titled Relation to Existing Prediction Models (line 568). This section summarises previous work to develop dementia risk models aimed at an older general population. We particularly focus on models that meet our study’s criteria of being amenable to automation in UK primary care and that do not include cognition/memory impairment factors. We find only three such models, one of which is the DRS while the other two are small (7 and 2 factors) and have low external validity. More generally, a study that evaluated the external validity of 10 models without cognitive factors, found none to have a C-statistic above 0.73. We feel this adds justification to a search for better performing models, either by discovery of novel predictors or through larger models that can capture more of the differences between patients that contribute to their overall risk.*

*Notwithstanding this, to our minds the strongest evidence supporting our models are the points we make in the paper that: (1) all of our factors were prespecified on the basis of possessing an evidence base and only included in our final models if their direction of effect was in line with the literature; (2) the great majority of the included factors also feature in more than one previously published dementia prediction model. (3) our sample size analysis indicated that we had sufficient data to build models with up to 200 factors whilst keeping overfitting and other biases within acceptable limits.*
